# Chronic Pressure Overload Results in Deficiency of Mitochondrial Membrane Transporter ABCB7 Which Contributes to Iron Overload, Mitochondrial Dysfunction, Metabolic Shift and Worsens Cardiac Function

**DOI:** 10.1038/s41598-019-49666-0

**Published:** 2019-09-11

**Authors:** Vikas Kumar, Aneesh Kumar A., Rahul Sanawar, Abdul Jaleel, T. R. Santhosh Kumar, C. C. Kartha

**Affiliations:** 1 Cardiovascular Diseases and Diabetes Biology, Rajiv Gandhi Centre for Biotechnology (RGCB), Trivandrum, Kerala India; 2 Cancer Research Program, Rajiv Gandhi Centre for Biotechnology (RGCB), Trivandrum, Kerala India; 3 Graduate studies, Manipal Academy of Higher Education (MAHE), Manipal, Karnataka India

**Keywords:** Metabolomics, Cardiac hypertrophy

## Abstract

We examined the hitherto unexplored role of mitochondrial transporters and iron metabolism in advancing metabolic and mitochondrial dysfunction in the heart during long term pressure overload. We also investigated the link between mitochondrial dysfunction and fluctuation in mitochondrial transporters associated with pressure overload cardiac hypertrophy. Left ventricular hypertrophy (LVH) was induced in 3-month-old male Wistar rats by constriction of the aorta using titanium clips. After sacrifice at the end of 6 and 15 months after constriction, tissues from the left ventricle (LV) from all animals were collected for histology, biochemical studies, proteomic and metabolic profiling, and gene and protein expression studies. LV tissues from rats with LVH had a significant decrease in the expression of ABCB7 and mitochondrial oxidative phosphorylation (mt-OXPHOS) enzymes, an increased level of lipid metabolites, decrease in the level of intermediate metabolites of pentose phosphate pathway and elevated levels of cytoplasmic and mitochondrial iron, reactive oxygen species (ROS) and autophagy-related proteins. Knockdown of ABCB7 in H9C2 cells and stimulation with angiotensin II resulted in increased ROS levels, ferritin, and transferrin receptor expression and iron overload in both mitochondria and cytoplasm. A decrease in mRNA and protein levels of mt-OXPHOS specific enzymes, mt-dynamics and autophagy clearance and activation of IGF-1 signaling were also seen in these cells. ABCB7 overexpression rescued all these changes. ABCB7 was found to interact with mitochondrial complexes IV and V. We conclude that in chronic pressure overload, ABCB7 deficiency results in iron overload and mitochondrial dysfunction, contributing to heart failure.

## Introduction

Adenosine triphosphate (ATP) binding cassette transporters (ABC transporters) are membrane proteins concerned with the exchange of a wide spectrum of small inorganic molecules, substrates, metabolites, metabolic end products, signaling molecules and drugs across membranes of cellular organelles and cells^1–3^. ABC transporters are most abundantly expressed in organs such as the heart with a high metabolic rate. The transporters ABCB6, ABCB7, ABCB8 and ABCB10 which belong to the half transporter B subfamily (ABCB group) are localized to the mitochondria^4^. ABCB6 is reported to localize in the plasma membrane, red blood cell membrane, melanosomes, mitochondria, and endolysosomes^5–10^. Mitochondrial ABC transporters along with mitoferrin (iron importer) and frataxin (storage/iron sensing regulator) control iron uptake and utilization in mitochondria. These transporters control mitochondrial electron transport chain (ETC) function, iron homeostasis, and levels of reactive oxygen species (ROS) in cells^2–4,11–14^. ABCB6 regulates porphyrin transport^15^. Our current knowledge on ABCB7 function remains presumptive^11–14,16–26^. ABCB7 function in the heart is also not known.

In recent times, there is a growing interest in the link among mitochondrial ABC transporters, iron metabolism, and cardiovascular diseases^2–4,16–29^. The heart is a highly metabolically active organ which requires a continuous supply of ATP to sustain its contractile function. A robust supply of iron is necessary to support the vigorous metabolic activity of the heart. Disturbances in iron metabolism are recognized to have pathologic consequences resulting in cardiomyopathy and aggravating clinical outcomes in heart failure^14,29–32^.

The role of mitochondrial transporters and cardiac iron metabolism in advancing metabolic and mitochondrial dysfunction in the heart during chronic pressure overload has not been deeply explored. In this report, we demonstrate that there is a decreased expression of ABCB7 in the heart subjected to chronic pressure overload. We also provide evidence to indicate that deficiency of this mitochondrial membrane transporter leads to mitochondrial iron overload and associated metabolic alterations, increases ROS in the mitochondria, alters mitochondrial OXPHOS function, mt-dynamics and impairs autophagy, all of which could contribute to the development of cardiac failure in the hypertrophic heart.

## Results

### Cardiac hypertrophy secondary to chronic pressure overload is associated with mitochondrial dysfunction, metabolic shift, and down-regulation of ABCB7

**Figure 1:**
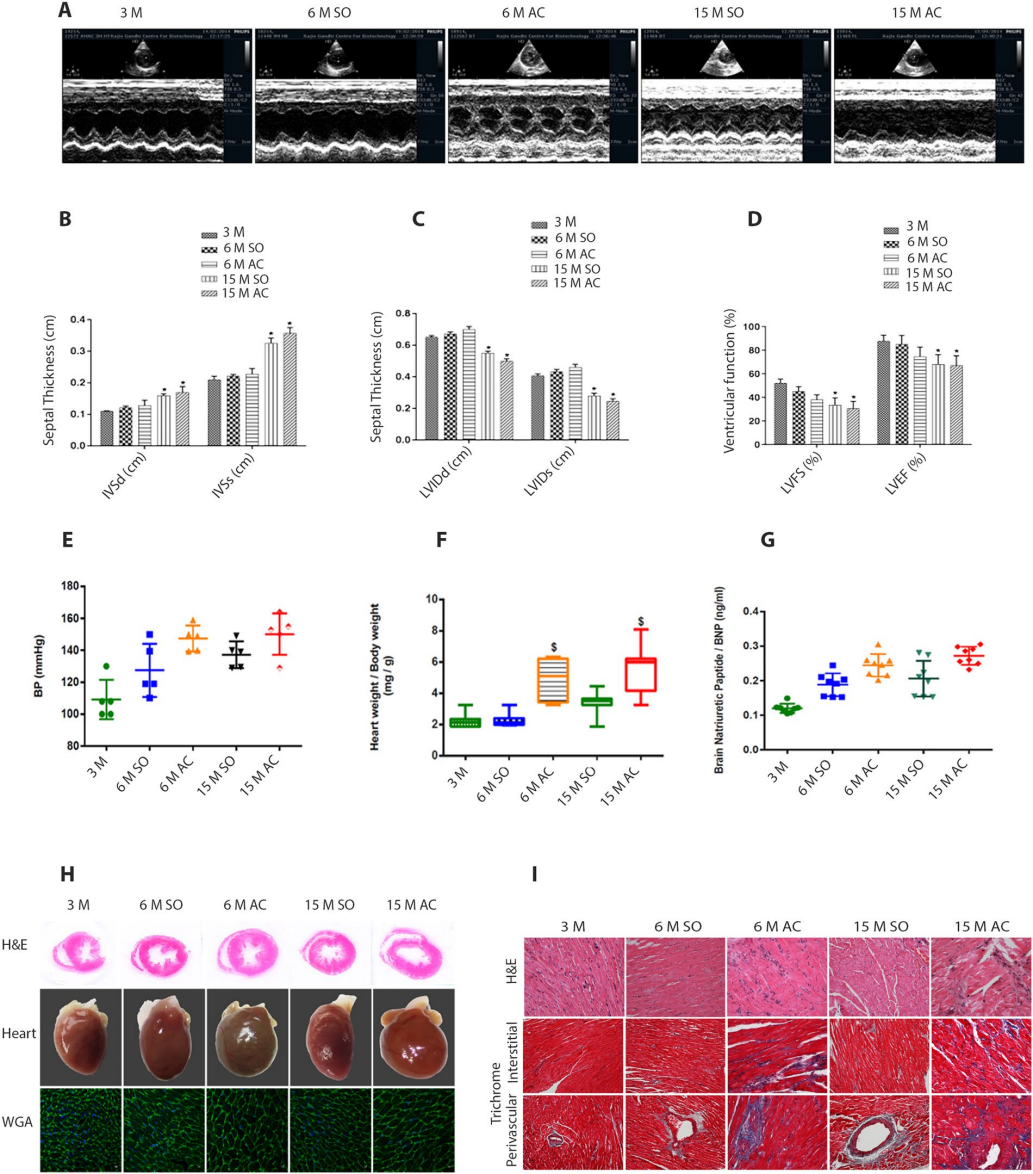
Cardiac structure and function in rats which had aortic constriction (AC) after 6 and 15 months when compared with sham-operated (SO) and 3-month-old rats. (**A**) Representative M-Mode echocardiographic images. Left ventricular hypertrophy is seen in rats with aortic constriction at the end of 6 months (6M AC) and left ventricular dilatation is seen in rats with aortic constriction at the end of 15 months (15M AC). (**B**–**D**) Left ventricular septal thicknesses, internal dimensions, fractional shortening, and ejection fraction in different groups. *P < 0.05, AC vs 3M and SO; (n = 8). (**E**–**G**) Blood pressure (BP), heart weight/body weight ratio, and serum brain natriuretic peptide (BNP) levels in the animals; (n = 8). (**H**) Representative photomicrograph of hematoxylin & eosin (top panel) and wheat germ agglutinin (lower panel; scale bar, 5 μm) stained cross sections of the heart and whole hearts (middle panel; scale bar, 10 mm). (**I**) Representative photomicrograph of Masson’s trichrome stained cross sections of the hearts. Blue stained areas denote fibrosis. Data are expressed as mean ± SEM. 3M, 3 months; 6M SO, 6 months after sham operation; 6M AC, 6 months after aortic constriction; 15M SO, 15 months after sham operation; 15M AC, 15 months after constriction; SO, Sham operation; AC, Aortic constriction.

Rats which had constriction of the aorta at 3 months of age developed left ventricular hypertrophy after 6 months of aortic constriction (AC). Rats, sham-operated (SO) at 3-month of age did not have left ventricular hypertrophy after 6 months of sham operation. Severe left ventricular hypertrophy was seen at the end of 15 months in rats which had constriction of the aorta. Some of the animals in this group had left ventricular dilatation and decline in left ventricular ejection fraction as well (Fig. 1A–D). We did not observe a significant change in blood pressure among different groups of rats (Fig. 1E). Heart weight/body weight ratio was increased in all animals which underwent aortic constriction (Fig. 1F). Brain natriuretic peptide (BNP) levels were elevated in the serum of rats with cardiac hypertrophy, both at 6 and 15 months after constriction of the aorta (Fig. 1G). Histology revealed cardiomyocyte hypertrophy and myocardial fibrosis in rats which underwent constriction of the aorta (Fig. 1H,I, and Supplementary Fig. S2A–C).

**Figure 2:**
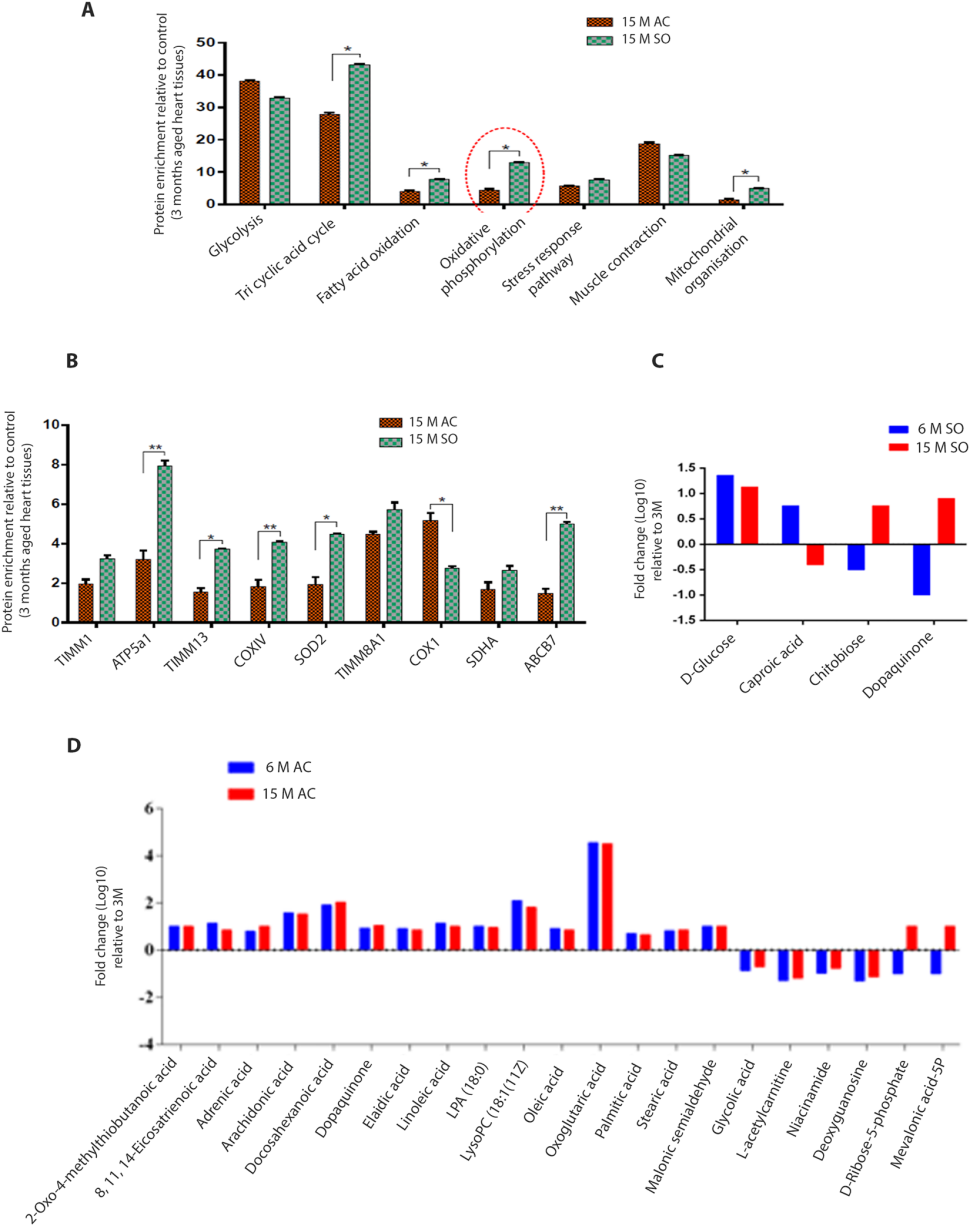
Downregulation in fatty acid metabolism pathway and accumulation of fatty acid/lipid metabolites were observed in left ventricular tissues of rats which underwent aortic constriction when compared to sham-operated and 3-month-old rats. (**A**) Results of Proteomic profiling/LC-MS of heart tissues. A decrease in proteins of fatty acid metabolism and oxidative phosphorylation pathway is seen in tissues from rats after 15 months of aorta constriction. *P < 0.05, 15M AC vs. 15M SO; (No. of rats in each group = 4). (**B**) Results of LC-MS analysis. A decrease in expression of proteins involved in the mitochondrial organization is observed. *P < 0.05, 15M AC vs 15M SO; n = 4. (**C,D**) Results of metabolic profiling/LC-MS analysis. Enrichment of fatty acid and lipid metabolites is seen in heart tissues from aorta constricted rats when compared with sham-operated and 3-month-old rats. *P < 0.05, 6M AC vs. 15M AC and 6M SO vs 15M SO; n = 8. Data are expressed as mean ± SEM. 3M, 3 months; 6M SO, 6 months after sham operation; 6M AC, 6 months after aortic constriction; 15M SO, 15 months after sham operation; 15M AC, 15 months after constriction; SO, Sham operation; AC, Aortic constriction.

**Figure 3:**
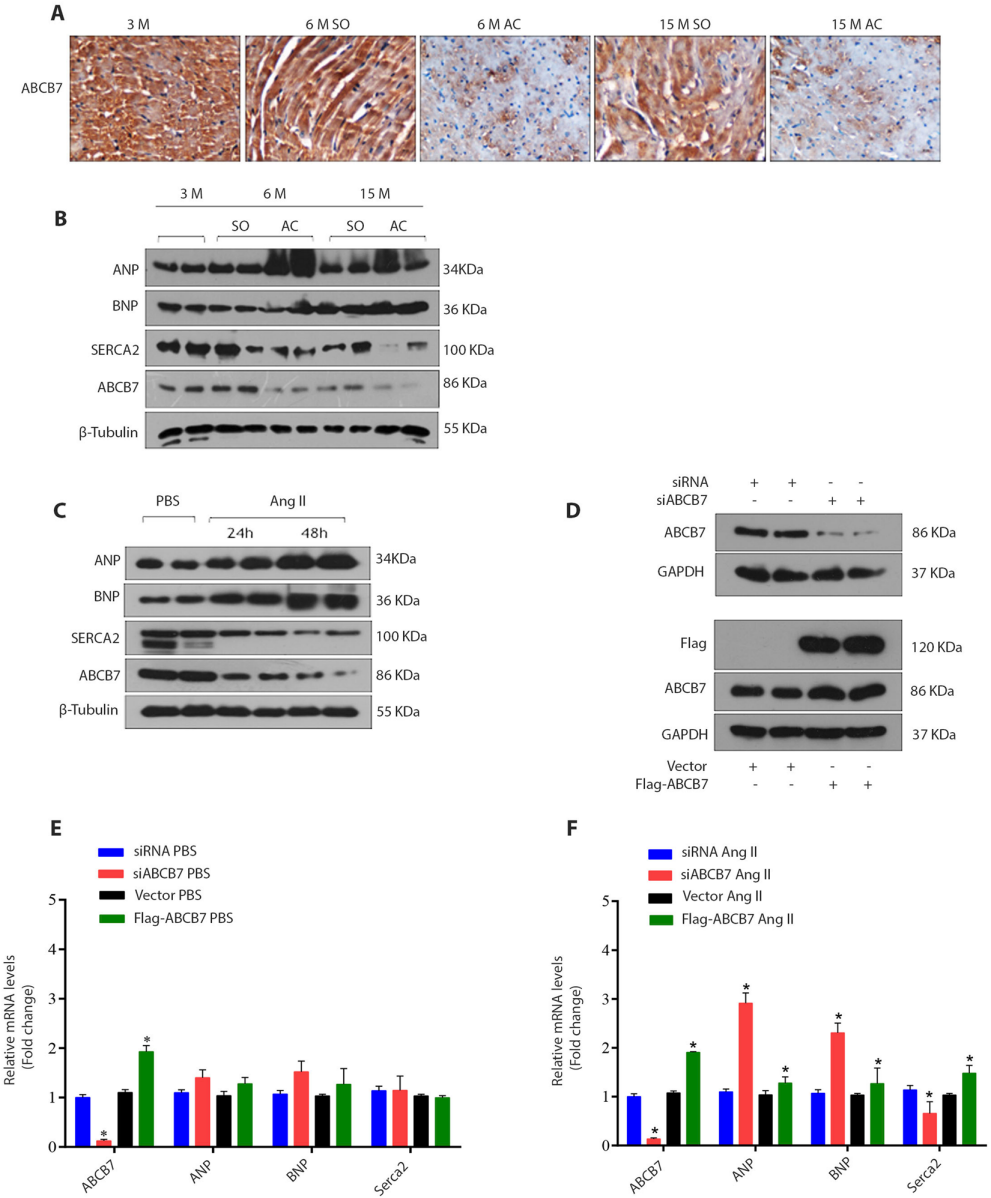
ABCB7 is downregulated in hypertrophied ventricles and cardiomyocytes with ABCB7 deficiency and stimulated with angiotensin II. (**A**) Photomicrograph of cross sections of the heart probed with ABCB7 antibody. A decrease in ABCB7 expression is seen in ventricles of rats both at 6 and 15 months after aorta constriction. (**B**) Western blots of ventricular tissues from rats with constriction of aorta, sham-operated and 3-month-old rats. (**C**) Western blots of lysates of H9C2 cells treated with AngII (1 μM) for 24 and 48 hours and analyzed for ABCB7 protein expression. (**D**) Representative image of ABCB7 and Flag expression in H9C2 cells transfected with siABCB7 or Flag-ABCB7 (n = 3 independent experiments). (**E**,**F**) Results of mRNA expression of hypertrophy markers in H9C2 cells transfected with either siABCB7 or flag-ABCB7 and stimulated with angiotensin II for 24 hours. *P < 0.05, siABCB7 vs control siRNA and vector vs flag-ABCB7; n = 4. Data are expressed as mean ± SEM. AngII, Angiotensin II.

**Figure 4:**
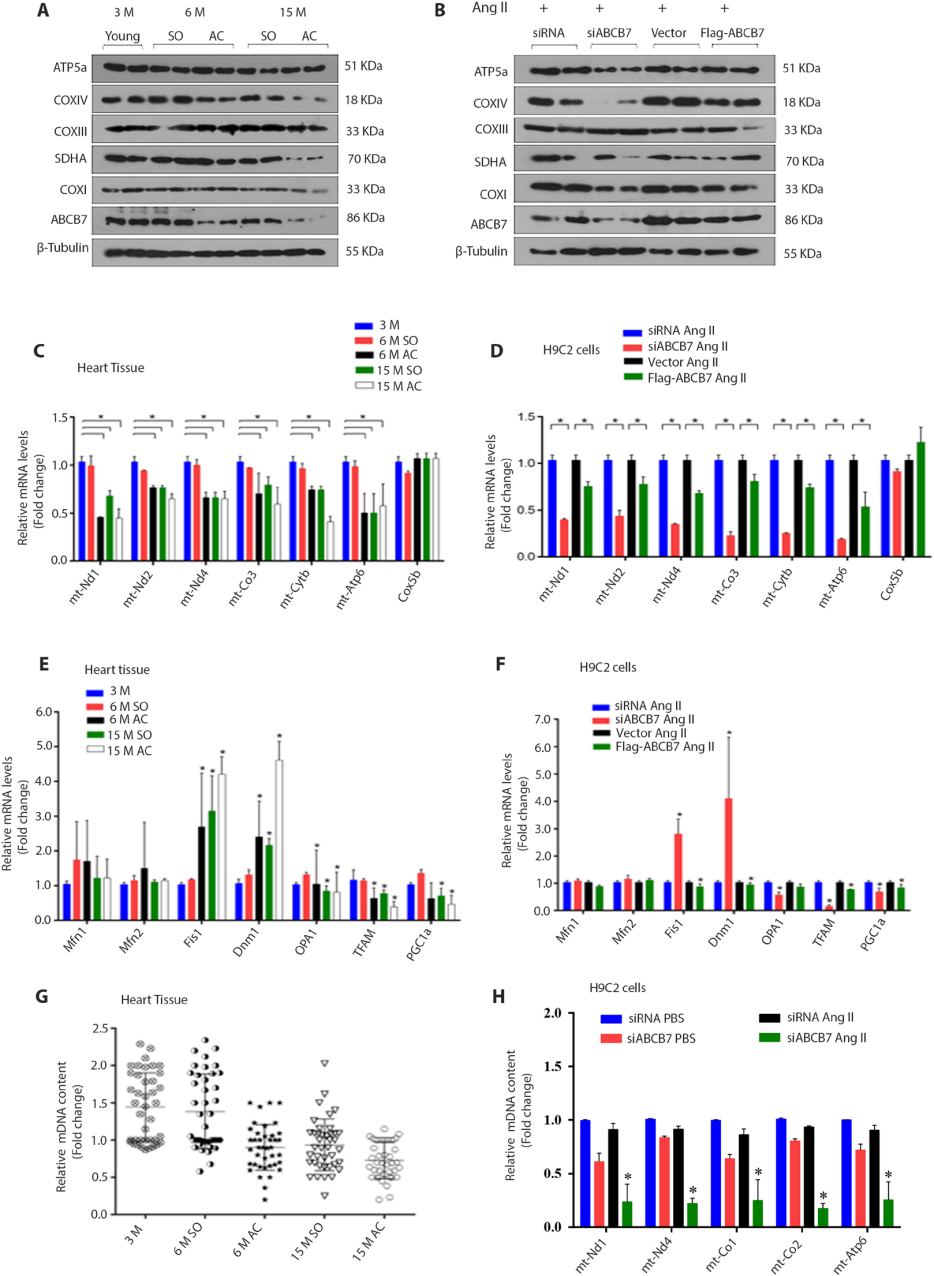
Myocardial ABCB7 regulates mitochondrial biogenesis. (**A**) ABCB7 and mitochondrial OXPHOS complex proteins expression from heart tissue of rats (n = 4, each lane represents an experiment on cardiac tissue from one rat). (**B**) Western blots of H9C2 cells transfected with either siABCB7 (100 nM) or Flag-ABCB7 and stimulated with angiotensin II and then analyzed for expression of ABCB7 and mitochondrial OXPHOX complex proteins. (**C**,**D**) Results of mRNA expression analysis of mitochondrial and nuclear-encoded genes in heart tissues of rats (*P < 0.05, AC vs 3M and SO; n = 4) and H9C2 cells stimulated with angiotensin II after transfection with either siABCB7 or Flag-ABCB7. *P < 0.05, siABCB7 vs control siRNA and vector vs flag-ABCB7; n = 4. (**E**,**F**) Results of mRNA expression analysis for genes which regulate mitochondrial dynamics in heart tissues of rats (*P < 0.05, AC vs 3M or SO; n = 4) and H9C2 cells stimulated with angiotensin II after transfection with either siABCB7 or Flag-ABCB7. *P < 0.05, siABCB7 vs control siRNA and vector vs flag-ABCB7; n = 4. (**G**,**H**) Results of qPCR analysis for cardiac mitochondrial DNA content in heart tissue of rats. Dots represent qPCR results related to 5 pairs of primers from ventricular samples from each group of animals (*P < 0.05, AC vs 3M and SO; n = 4) and H9C2 cells transfected with either siABCB7 or Flag-ABCB7. *P < 0.05, siABCB7 vs control siRNA and vector vs flag-ABCB7; n = 4. Data are expressed as mean ± SEM. OXPHOS, Oxidative phosphorylation.

We did an unbiased LC-MS analysis of samples from cardiac left ventricles from 3-month-old, sham-operated animals and rats which had constriction of the aorta and developed left ventricular hypertrophy (4 samples from each group). Proteomic profiling suggested downregulation in fatty acid oxidation (FAO) and oxidative phosphorylation pathways and unaltered glycolysis pathway in the hearts of rats with left ventricular hypertrophy. These changes were not observed in the hearts of sham-operated and 3-month-old rats (Fig. 2A). Proteomic profiling of hypertrophic hearts also revealed a significant down-regulation in the expression of ABCB7, COXIV, ATP5a1, TIMM1, TIMM13, and SOD2, all of which are proteins that regulate mitochondrial function (Fig. 2B and Supplementary Fig. S3A,B). These results were confirmed by qRT-PCR, Western blot, and immunohistochemistry (IHC) analysis (Figs 3 and 4). Decreased expression of enzymes regulating FAO and OXPHOS pathways in the hearts of rats with cardiac hypertrophy suggests a switch in fuel source from FAO to glycolysis. To confirm the metabolic switch and its progression at the level of metabolites, we performed metabolomics (LC-MS) analysis of left ventricular samples obtained from the three groups of rats. We observed an enrichment of unsaturated fatty acids (oleic acid, linoleic acid, elaidic acid, arachidonic acid, docosahexanoic acid), saturated fatty acids (myristic acid, palmitic acid, stearic acid, caproic acid), phospholipids (lysophosphatidylcholine/Lyso-PC, phosphatidylethanolamine/PE, lysophosphatidic acid/LPA) and other metabolites which act as intermediates in fatty acid transport or metabolism (stearoylcarnitine, 2-Oxo-4-methylthiobutanoic acid) in the hearts of rats which had constriction of aorta (after 6 and 15 months) when compared with 3-month-old and sham-operated animals. Decreased enrichment of acetyl carnitine, malonic semi-aldehyde which are involved in fatty acid transport was observed in the hearts of rats which had constriction of the aorta (after 6 and 15 months) when compared with 3-month-old and sham-operated animals (Fig. 2C,D; Supplementary Figs S2, S3C–F and Tables 1–6). There were enhanced levels of D-Ribose-5-phosphate (intermediary metabolites of pentose phosphate pathway) and mevalonic acid-5-phosphate (intermediate metabolites in cholesterol synthesis) in left ventricular tissues of rats 15 months after aorta constriction when compared with heart tissues from rats 6 months after constriction (Fig. 2C,D; Supplementary Fig. S2 and Tables 1–6).

### ABCB7 is down-regulated in hearts with pressure-overload hypertrophy

Cardiac ABCB7 expression was significantly lower in rats with pressure overload-induced hypertrophy when compared with 3-month-old and sham-operated rats (Fig. 3A,B, and Supplementary Fig. S4D,E). Expression of ABCB7 was significantly decreased in H9C2 cells after stimulation with angiotensin II for 24 hours as well, and the expression further reduced after 48 hours (Fig. 3C and Supplementary Fig. S4F). ABCB6 and ABCB8 expressions were significantly higher in the hearts of 6 months aorta constricted and sham-operated rats, 15 months sham-operated rats and were lower in the hearts of 15 months aorta constricted rats when compared with 3-month-old rats. ABCB10 expression was similar in aorta constricted and sham-operated rats and was significantly increased in both aorta constricted and sham-operated rats when compared with 3-month-old rats (Supplementary Fig. S5A). Increased expression of ABCB6, ABCB8, and ABCB10 were observed in the ABCB7 silenced H9C2 cells (Supplementary Fig. S5B).

### **A**BCB7 deficiency can exaggerate cardiac hypertrophy

To test the role of ABCB7 in cardiac hypertrophy, we transfected H9C2 cells with ABCB7- targeting siRNA to downregulate ABCB7 or with flag-ABCB7 to induce ABCB7 overexpression and then stimulated the cells with angiotensin II to induce hypertrophy (Fig. 3D and Supplementary Fig. S5C,D). We observed that knockdown of ABCB7 resulted in an exaggerated hypertrophy response, whereas overexpression of ABCB7 rescued the cells from cardiac hypertrophy (Fig. 3E,F). ABCB7 silencing resulted in increased expression of ANP, BNP, and decrease in Serca2 while ABCB7 overexpression attenuated the expression of ANP, BNP, and Serca2 (Fig. 3E,F). These results suggest that loss of ABCB7 could contribute to intensifying cardiomyocyte hypertrophy.

### ABCB7 deficiency promotes mitochondrial dysfunction in hypertrophic cardiomyocytes

The LC-MS analysis of left ventricular tissues from rats with cardiac hypertrophy revealed a decreased expression of mitochondrial proteins (Fig. 2B). We observed a reduced expression of proteins of mitochondrial OXPHOS complex and decreased mRNA expression of mitochondrial encoded genes in rats with aorta constriction when compared with 3-month-old and sham-operated rats (Fig. 4A,C, and Supplementary Fig. S5E). The mRNA levels of Opa1, TFAM, and PGC1a genes were significantly lower (*P* < 0.05) and Fis1 and Dnm1 genes were overexpressed in rats with pressure-overload hypertrophy when compared with 3-month-old and sham-operated rats (Fig. 4E). The qPCR analysis confirmed a decrease in mitochondrial genomic DNA content (approximately 2 fold) in rats with aorta constriction when compared with 3-month-old and sham-operated rats after 6 and 15 months (Fig. 4G)_._

We used H9C2 cells to test whether ABCB7 regulates mitochondrial function in these cells. Expression of mitochondrial OXPHOS complex proteins was decreased after silencing ABCB7 in cells stimulated with angiotensin II. ABCB7 overexpression increased or reinstated the expressions (Fig. 4B and Supplementary Fig. S5F). The mRNA expressions of mitochondrial encoded genes were also decreased after silencing ABCB7 gene in H9C2 cells, and ABCB7 overexpression reinstated mRNA expression of these genes (Fig. 4D). Silencing of ABCB7 also resulted in a decreased expression of Opa1, TFAM, PGC1a, and overexpression of Fis1 and Dnm1 genes in H9C2 cells. Expressions of Mfn1 or Mfn2 genes were unaltered (Fig. 4F). The decrease in mitochondrial genomic DNA content in ABCB7 deficient cells stimulated with angiotensin II was seen in the analysis (Fig. 4H).

### ABCB7 deficiency in cardiac hypertrophy is also associated with iron overload

ABCB7 is required for the formation of Fe-S cluster proteins and heme biosynthesis in cells. ABCB7 deficiency is known to result in increased iron overload and impaired transport of iron across the mitochondrial membrane to the cytosol^11–14,18,19^. We measured serum iron levels in rats with cardiac hypertrophy and did not find significant changes when compared to 3-month-old and sham-operated rats (Supplementary Fig. S6A). In contrast to this, we observed an elevated iron level in the heart tissue of rats which underwent aorta constriction and had hypertrophy when compared with heart tissues from 3-month-old and sham-operated rats (Supplementary Fig. S6B). Perl’s Prussian blue staining also revealed iron deposits in the heart tissues of rats which had left ventricular hypertrophy, at the end of 6 months after constriction of the aorta. Iron positivity was not seen in heart tissues from 3-month-old and at the end of 6 months in sham-operated rats (Supplementary Fig. S6C). Iron deposits were also seen in the hearts of rats, 15 months after aorta constriction as well as in sham-operated rats and had age-related cardiac hypertrophy, 15 months after sham operation. Increase in expression of iron regulatory proteins TF, Tfrc, Fth-1, and Ftl-1 were also observed in heart tissues of rats which underwent constriction of the aorta and had cardiac hypertrophy (Supplementary Fig. S6D). ABCB7 silencing in H9C2 cells under angiotensin II stimulation resulted in the enhanced expression of iron regulatory proteins. ABCB7 overexpression reinstated the expression of these genes in H9C2 cells (Supplementary Fig. S6E). We also found an elevated level of iron in both mitochondrial and cytoplasmic fractions of ABCB7 deficient cells (Supplementary Fig. S6F,G). To understand the significance of ABCB7 silencing mediated iron overload in cardiac hypertrophy, we had given deferiprone (an iron chelator) treatment to H9C2 cells transfected with either siRNA or siABCB7. We did not observe a significant change in expression of hypertrophy markers in H9C2 cells transfected with siABCB7 and treated with deferiprone. In addition, there was no significant change in the expression of iron regulatory genes (TF, FTH1, FTL1) in H9C2 cells treated with deferiprone when compared with cells transfected with siABCB7 (Supplementary Fig. S6H).

### **I**ron overload in hypertrophic cardiomyocytes leads to oxidative stress

Iron overload in ABCB7 deficient cells could contribute to ROS generation. ROS in cells act as second messengers and activate protective signaling mechanisms^32–36^. An increase in H_2_O_2_ level was observed in heart tissue of rats which underwent aorta constriction when compared with 3-month-old and sham-operated rats (Supplementary Fig. S7A). We transfected H9C2 cells with different concentrations of siABCB7 (100 nM or 200 nM). MitoSOX Red and H_2_O_2_ assay were then performed for measurement of ROS generation in cells. H_2_O_2_ assay revealed increased H_2_O_2_ levels in H9C2 cells after silencing ABCB7 using siRNA (Supplementary Fig. S7B,C). After transfection with 200 nM concentration of ABCB7 siRNA, H_2_O_2_ levels were further increased in H9C2 cells. A comparable degree of increase in H_2_O_2_ levels was seen in antimycin-A treated H9C2 cells. In MitoSOX Red assay, an increase in fluorescence intensity was seen after ABCB7 silencing (100 nM). Fluorescence intensity was more in the cells after transfection with 200 nM concentration of ABCB7 siRNA (Supplementary Fig. S7D). FACS analysis also confirmed MitoSOX positive H9C2 cells transfected with siABCB7 (Supplementary Fig. S7E). A decrease in SOD2, catalase, and an increase in expression of NFkB protein were seen in H9C2 cells when stimulated with angiotensin II after ABCB7 silencing (Supplementary Figs S7F and S5G). Overexpression of ABCB7 reinstated the expression of the antioxidant genes (Supplementary Figs S7G and S5H). These findings provide a basis for enhanced ROS and H_2_O_2_ levels in H9C2 cells after silencing of the ABCB7 gene. Mitochondrial membrane potential assessed using TMRM staining was impaired after transfection of H9C2 cells at both 100 and 200 nM concentrations of ABCB7 siRNA (Supplementary Fig. S8A,B). Altogether, these results indicate that reduced ABCB7 gene expression associated with cardiac hypertrophy can affect an increase in iron and H_2_O_2_ levels, ROS generation, and loss of mitochondrial membrane potential in H9C2 cells.

### ABCB7 deficiency impairs autophagy in hypertrophic cardiomyocytes

To examine whether ABCB7 loss associated with hypertrophy, contributes to enhanced accumulation of damaged mitochondria or impaired autophagy clearance, we checked autophagy in left ventricular tissues of rats which underwent aorta constriction and developed left ventricular hypertrophy. A decrease in the conversion of LC3 I to LC3 II, a hallmark of autophagy inhibition was observed in heart tissues of rats which underwent aorta constriction. Decreased expression of beclin-1 and increased expression of p62 proteins were also observed in heart tissues of rats which had aortic constriction (Supplementary Fig. S9A,B). To confirm whether ABCB7 deficiency mediates impaired autophagy in cardiomyocytes, we transfected H9C2 cells using either siABCB7 or Flag-ABCB7 in the presence or absence of carbonyl cyanide-4-(trifluoromethoxy) phenylhydrazone (FCCP, 20 μmol/liter), a mitochondrial uncoupling agent and an autophagy inducer. Over-expression of ABCB7 resulted in LC3 II accumulation in H9C2 cells, and ABCB7 silencing reduced the expression of LC3 II, which was not restored in the presence of FCCP (Supplementary Fig. S9C,D). Bafilomycin A1 (BFA, 100 nmol/liter), is an inhibitor of late-stage autophagy and promotes accumulation LC3 II and thus could be used to assess autophagy flux. Treatment of ABCB7 silenced H9C2 cells with BFA also did not restore the reduced expression of LC3II (Supplementary Fig. S9E,F). The findings imply that in ABCB7 deficient cardiomyocytes, the autophagy process is impaired.

### ABCB7 regulates IGF-1 signaling in hypertrophic cardiomyocytes

**Figure 5:**
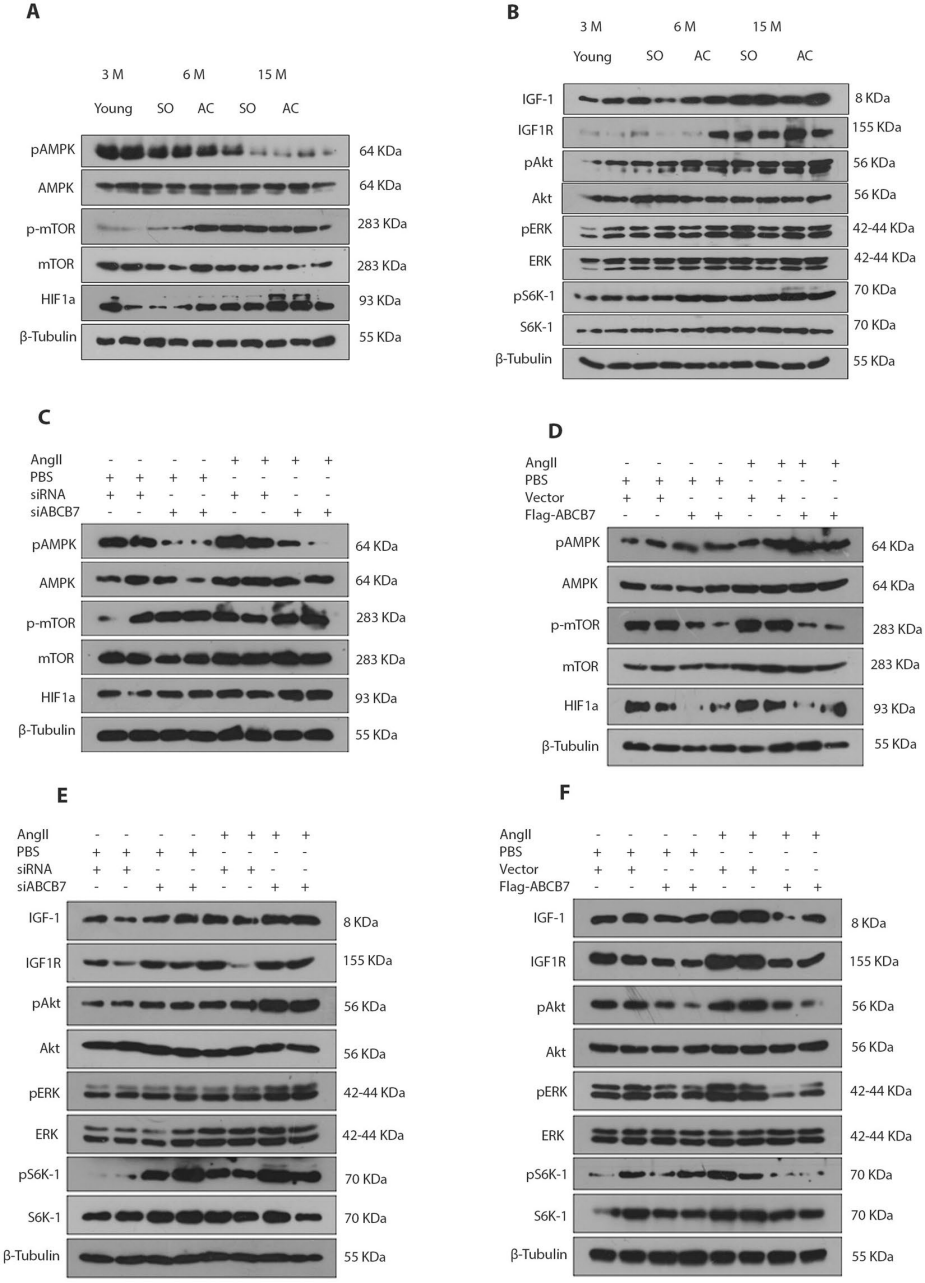
ABCB7 deficiency impairs iron balance and catalyzes ROS generation, which in turn activates IGF-1/HIF1α signaling in cardiomyocytes. (**A**,**B**) Representative Western blots for HIF1α, p-mTOR and pAMPK and IGF-1 signaling gene expression in heart tissues of 3 months old, sham-operated and aorta constricted rats. (**C,D**) Representative Western blots for HIF1α, p-mTOR, and pAMPK gene expression in H9C2 cells transfected with siABCB7 or control siRNA or with control vector or flag-ABCB7, followed by stimulation with AngII or PBS for 24 hours. (**E**,**F**) Representative Western blots for expression of IGF-1 signaling genes in H9C2 cells transfected with siABCB7 or control siRNA or with control vector or flag-ABCB7, followed by stimulation with AngII or PBS for 24 hours.

Earlier studies have revealed that IGF-1/HIF1α pathway is activated during iron overload and ROS burst, in cardiac hypertrophy and aging conditions^36–38^. We analyzed the expression of IGF1R, IGF-1, pAkt (Ser 473), pERK (Thr 202/Tyr 204) and pS6K (Thr 389) genes, the regulatory genes of Insulin/IGF-1 signaling pathway in left ventricular tissues of aorta constricted rats. An upregulation of these proteins was seen at both 6 months and 15 months after the constriction. There was also a decrease in pAMPK (Thr 172) and an increase in p-mTOR (Ser 2448) and HIF1α protein expression in hypertrophic hearts (Fig. 5A,B and Supplementary Fig. S10A–H). We examined whether downregulation of ABCB7 can activate IGF-1/HIF1a signaling mechanisms in hypertrophic cardiomyocytes. After transfection with siABCB7 or flag-ABCB7, H9C2 cells were stimulated with angiotensin II for 24 hours and analyzed the expression of proteins of HIF1a and IGF-1 pathway. A decrease in pAMPK and an increase in p-mTOR and HIF1α protein expression were also seen in H9C2 cells after ABCB7 silencing. ABCB7 over expression reinstated their expression. (Fig. 5C,D, and Supplementary Fig. S11A,B). Western blot analysis revealed an increase in the expression of IGF1R, IGF-1, pAkt, pERK and pS6K genes in H9C2 cells silenced with siABCB7 and ABCB7 overexpression normalized the expression of these proteins in H9C2 cells (Fig. 5E,F, and Supplementary Fig. S11C,D). The mRNA expression analysis also revealed an upregulation in ACC1, PGK1, and Akt (S473) expression in ABCB7 silenced H9C2 cells (Supplementary Fig. S11F). An upregulation in ACC1 and Akt (S473) protein expression was observed in ABCB7 silenced hypertrophic H9C2 cells. ABCB7 overexpression attenuated the expression of these proteins in hypertrophic H9C2 cells (Supplementary Fig. S12). To investigate the significance of ABCB7 silencing mediated IGF1R signaling axis in cardiac hypertrophy or mitochondrial dynamics, we silenced the IGF1R gene in H9C2 cells transfected with either siRNA or siABCB7. IGF1R silencing significantly increased ABCB7 expression in H9C2 cells but did not significantly change the expression of ABCB7 in H9C2 cells transfected with siABCB7. Decreased in the expression of hypertrophy markers (ANP, BNP) in H9C2 cells transfected with both siABCB7 and si-IGF1R were also observed. We observed that expression of Fis1 was unregulated and PGC1a gene, in contrast, was down-regulated in H9C2 cells transfected with either siABCB7 or si-IGF1R alone or in combination. Also, there was no significant change in expression of Dnm1 gene in H9C2 cells transfected with both si-IGF1R and siABCB7 when compared with cells transfected with siABCB7 (Supplementary Fig. S11E).

### ABCB7 interacts with mitochondrial complex proteins

**Figure 6:**
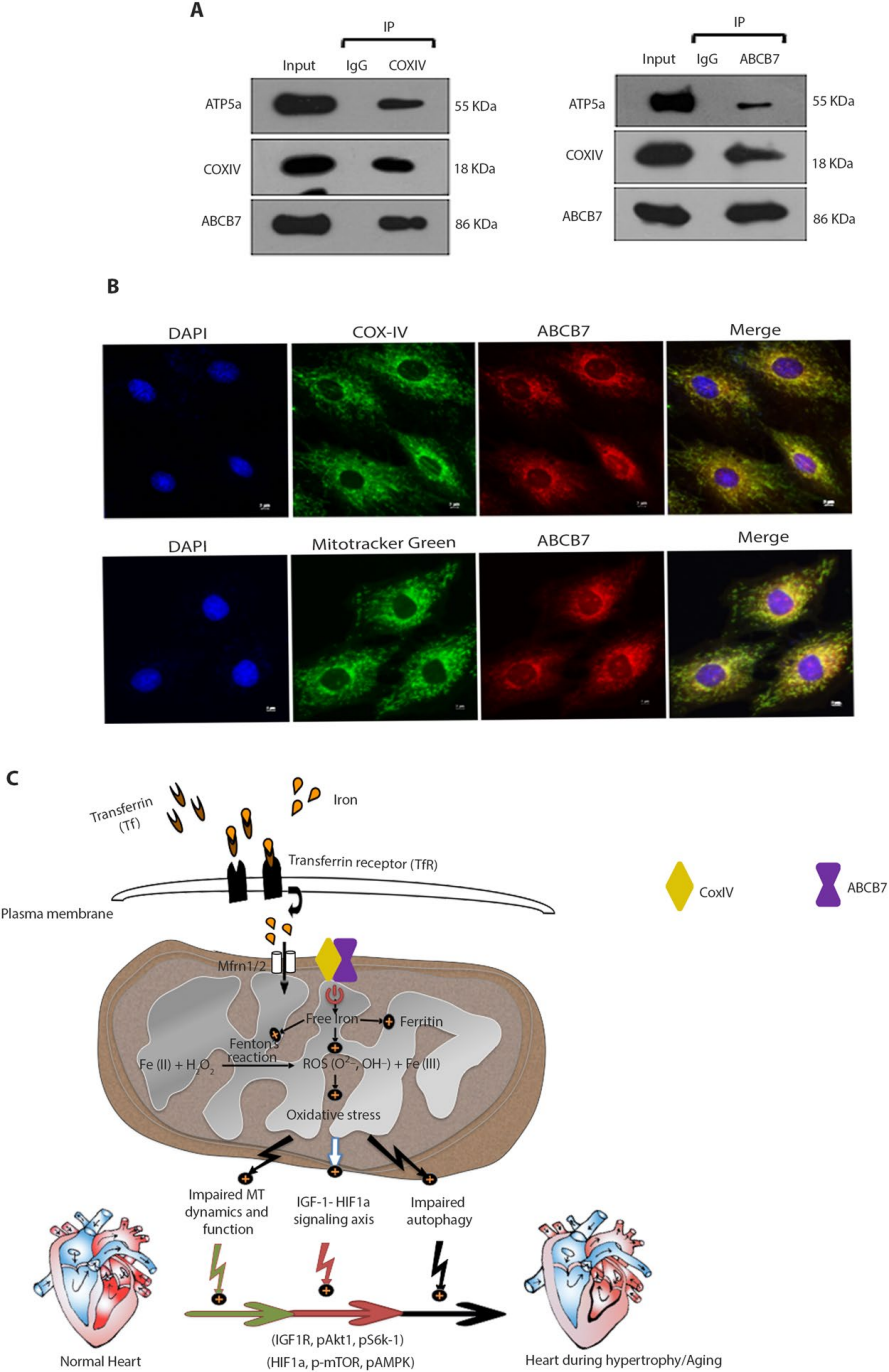
ABCB7 interacts and also co-localizes with mitochondrial complex IV protein. (**A**) Results of co-immunoprecipitation analysis for interaction between endogenous ABCB7 and mt-COXIV in H9C2 cells. H9C2 cell lysate was immunoprecipitated using ABCB7/COXIV antibody and analyzed by Western blotting. Images reveal that ABCB7 interacts with COXIV and COXV proteins of mitochondria. (**B**) Immunofluorescence images reveal that ABCB7 co-localizes with COXIV protein in H9C2 cells. (**C**) Schematic diagram of the role of ABCB7 in the heart and the proposed mechanisms which may promote cardiac dysfunction in chronic pressure overload. ABCB7 interacts with COXIV and regulates cellular iron homeostasis and cellular ROS levels in cardiomyocytes. Downregulation of ABCB7 results in impaired iron homeostasis, oxidative stress, mitochondrial dysfunction, altered autophagy, and activation of HIF1α-IGF1 signaling pathway in the hypertrophied heart. mt-COXIV, mitochondrial Complex IV; ROS, reactive oxygen species.

To determine the molecular interaction of ABCB7 with mitochondrial OXPHOS complex proteins, we performed co-immunoprecipitation (Co-IP) assays in H9C2 cells for mitochondrial complex proteins. Co-IP and reverse Co-IP suggested that ABCB7 interacts with COXIV (mitochondrial complex IV) and ATP5a (mitochondrial complex V) proteins but not with other mitochondrial electron transport chain proteins (Fig. 6A). Immunofluorescence analysis revealed a co-localization of ABCB7 with COXIV protein in H9C2 cells (Fig. 6B).

ABCB7’s co-regulatory proteins were identified using Co-Regulation Database (CORD) analysis, a meta-analysis program established for finding the target gene function. Proteins involved in the oxidative phosphorylation pathway, citrate cycle, and oxidative stress pathway were identified in CORD analysis as co-expressed and co-regulated with ABCB7 (Supplementary Excel File S2).

Our findings thus reveal that ABCB7 deficiency during chronic cardiac hypertrophy raise iron load in the heart, impairs mitochondrial homeostasis, and activates IGF-1-HIF1a signaling pathway (summarized in Fig. 6C).

## Discussion

Our studies in animal models and H9C2 cells have led to the novel discovery that sustained pressure load results in the deficiency of the mitochondrial ATP-binding cassette transporter ABCB7 in the hypertrophic heart. Our metabolic studies in cardiac tissues from pressure overload heart revealed alterations in glycolysis and fatty acid oxidation pathways. We also found evidence to indicate that deficiency of ABCB7 has associated with iron overload and oxidative stress in mitochondria as well as alterations in mitochondrial OXPHOS function and dynamics. ABCB7 interacts with mitochondrial OXPHOS proteins (COXIV and V). Our studies also suggest that ABCB7 regulates mitochondrial iron homeostasis, dynamics, and autophagy by modulating the IGF-1 signaling pathway in cardiomyocytes. ABCB7 could thus be involved in the modulation of transition from cardiac hypertrophy to failure. We have shown that ABCB7 silencing induces expression of markers of hypertrophy in cardiomyocytes. These markers are reduced on overexpression of ABCB7 in cells stimulated with angiotensin II to induce hypertrophy. Interestingly, expressions of ABCB6, ABCB8 and ABCB10 transporters were increased in the hypertrophic heart as well as in ABCB7 silenced H9C2 cells. These findings indicate the possible specific association of ABCB7 in pressure-overload cardiac hypertrophy.

ABCB7 is an inner mitochondrial membrane transporter that is highly expressed in muscle and regulates iron metabolism and mitochondria/cytosolic Fe–S cluster biogenesis^11–13,18,19^. The iron-sulfur clusters (ISC) are essential for the structural integrity and catalytic function of multiple mitochondrial enzymes. ISC and heme are components of ETC chain complexes I, II, III, and IV which drive the oxidative phosphorylation process culminating in ATP synthesis^1–4,16^. Deletion of ABCB7 in mice impairs Fe-S cluster protein biosynthesis and dysregulates hepatic iron metabolism. Fe-S clusters play an essential role in maintaining the structure and catalytic function of mitochondrial enzymes, especially the ETC complex I, II, III, and IV in cells^11–13,18,19^.

Recent studies suggest that elevated intracellular free iron level catalyzes the Fenton reaction and produces ROS and hydroxyl radicals in cells^33–36^. Our present study indicates that in pressure-overload hypertrophy, there is a deficiency of ABCB7 associated with raised levels of iron and iron-binding proteins as well as elevated levels of superoxide radical and hydrogen peroxide in the heart. Insulin/IGF1 and HIF1α are the components of key signaling mechanisms that regulate ROS levels associated with aging and cardiac hypertrophy and failure^36–38^. Studies in *Caenorhabditis elegans* have revealed that HIF1α and Insulin/IGF-1 signaling pathways act in concert to regulate stress resistance, levels of iron and iron-regulatory proteins^37^. We observed that IGF-1 and HIF1α signaling pathways are activated in ABCB7 deficient cardiomyocytes. Studies after si-IGF1R silencing in H9C2 cells revealed that IGF1R-pAKT1-pS6K1 signaling axis could be a mechanistic link between ABCB7 downregulation associated with cardiac hypertrophy and impaired mitochondrial function.

In ABCB7 deficiency, there is also a downregulation in the expression of electron transport chain (ETC) complex proteins I, II, IV, and V in the hearts with pressure-overload hypertrophy. These alterations could lead to impaired OXPHOS function, mitochondrial membrane integrity, and dynamics as well. Impaired mitochondrial OXPHOS pathway can lead to the production of ROS and mitochondrial dysfunction in cells^18,39,40^. An increase in ROS production in the mitochondria, as well as cytosol of cardiomyocytes, was found after ABCB7 silencing.

Our study thus indicates that ABCB7 is possibly vital for normal mitochondrial OXPHOS complex function in the heart and therefore, could have a significant role in regulating mitochondrial metabolism during cardiac remodeling associated with chronic pressure overload. Mitochondrial ABC transporter proteins such as ABCB6, ABCB8, and ABCB10 are already recognized to have a role in the regulation of iron level and Fe-S cluster proteins in cells^7–9,15,26–28^. Deletion of ABCB8, another mitochondrial transporter has been shown to lead to accumulation of ROS, impairment of iron export, and maturation of heme and iron-sulfur cluster proteins and contribute to cardiomyopathy in mice^27^. Heterozygous deletion of ABCB10 in the mice heart results in impaired mitochondrial bioenergetic function, increased oxidative damage, and reduced systolic and diastolic function after ischemia/reperfusion injury^28^. None of the ABC transporters has been previously shown to have a role in mitochondrial metabolism and mitochondrial dynamics/quality control mechanism in the heart subjected to chronic pressure overload.

A shift in mitochondrial substrate metabolism is recognized to be associated with progressive remodeling and failure in the aging heart. In the aging heart, there is a decrease in fatty acid oxidation and oxidative phosphorylation and increase in glycolysis pathways^41–44^. Metabolic remodeling in the aging heart contributes not only to impaired cardiac energetics but also to enhanced oxidative stress and heart failure^44–51^. Molecular basis for the progression and severity of altered mitochondrial metabolism during different stages of cardiac hypertrophy and evolution of cardiac failure in the hearts with pressure overload hypertrophic heart is still not completely delineated.

In our metabolic studies of the ventricle from old hearts subjected to chronic pressure overload, we found decreased expression of mitochondrial OXPHOS complex proteins, alterations in fatty acid oxidation and downregulation in oxidative phosphorylation pathways and significant alterations in the glycolysis pathway in the hypertrophic heart. Increased expression of fatty acid/lipid metabolites, decrease in acetylcarnitine and stearoyl carnitine (involved in fatty acid metabolism/transport) and decrease in expression of ribose 5 phosphate and mevalonic acid-5-phosphate (involved in pentose phosphate pathway and cholesterol biosynthesis) as observed in our metabolic studies suggest an impaired regulation of these pathways in the hearts of rats with severe pressure overload hypertrophy. The accumulation of fatty acid and metabolites of lipid metabolism were found to increase with the duration of pressure overload. In a recent study, overexpression of long-chain acyl-CoA synthetase in mice was found to lead to increased fatty acid uptake in the mitochondria of cardiomyocytes. This increased fatty acid uptake, in turn, resulted in palmitoyl-carnitine accumulation and an associated increase in ROS production. The authors of this study concluded that lipid overload resulted in mitochondrial dysfunction by altering the mitochondrial dynamics in cardiomyocytes^47,48^. Our findings are in concordance with previous reports that iron imbalance may impair the pentose phosphate pathway and cholesterol biosynthesis as well and determine metabolic fuel preference^52–54^. It has been suggested that increasing glycolysis coupled with glucose oxidation (OXPHOS) or increasing OXPHOS efficiency in the energy-starved failing heart could be a strategy to treat cardiac failure^44,52–55^.

Thus, we provide new insights into the significant role of the mitochondrial transporter protein ABCB7 in the pathogenesis of mitochondrial dysfunction and metabolic remodeling, which could contribute to the development of ventricular dysfunction in the hypertrophic heart. Our findings are significant, given that the role of mitochondrial transporters and cardiac iron metabolism in advancing metabolic and mitochondrial dysfunction in the heart subject to chronic pressure overload have not been deeply explored.

The limitations that can be raised in our study are that we have not used heterozygous ABCB7 knockout animals or primary cardiomyocytes. We also did not explore whether ABCB7 compared to the other ABCB-family members has any unique causative role in cardiac hypertrophy. Be that as it may, the new evidence from our experiments are sufficiently clear to indicate that chronic pressure overload results in ABCB7 deficiency in the hypertrophic heart and that the loss in ABCB7 function can contribute to secondary disturbances in iron homeostasis, mitochondrial function, and metabolism in the heart leading to worsening of cardiac function.

## Materials and Methods

### Reagents

ABCB7 siRNA (Sigma), antimycin A (Sigma), ADP (Sigma), rotenone (Sigma), carbonyl cyanide-*4*-(trifluoromethoxy) phenylhydrazone (FCCP), tetramethyl rhodamine, methyl ester, perchlorate (TMRM) (ThermoFisher Scientific), bafilomycin A1 (Sigma), angiotensin II (Sigma), Mito Sox Red (ThermoFisher Scientific), trichrome stain (Sigma), wheat germ agglutinin/WGA (Sigma), SYBR Green (Applied Biosystems), Amplex red hydrogen peroxide/peroxidase assay kit (Thermo Fisher Scientific), mitochondria isolation buffer (IBm1) composed of 1M Tris-HCl, 1M sucrose, 10% BSA, 1M EDTA, and 1M KCl. Mitochondria isolation buffer 2(IBm2) composed of 1M Tris-HCl, 1M sucrose, and 0.1M Tris-EGTA.

### Animal experiments and pressure overload-induced cardiac hypertrophy

All animal experiments were carried out after obtaining approval from the Institutional Animal Ethics Committee (IAEC) of Rajiv Gandhi Centre for Biotechnology, Trivandrum (Protocol No. IAEC/529/TRSK/2016). We strictly followed the rules and regulations of the Committee for Control for the Purpose of Control and Supervision of Experiments on Animals (CPCSEA), Government of India.

Three months old male Wistar rats were used for our experiments. Animals were maintained on a 12 hours day/light cycle in a temperature and humidity controlled room. The body weights of rats were recorded on a weekly basis. All animals were provided food and water *ad libitum* and observed for any untoward symptoms.

After an initial assessment of basal echocardiography parameters, 50 male Wistar rats (3 months old) were divided into three groups. Twenty rats (at the age of 3 months) underwent constriction of ascending aorta using titanium clip (aortic constricted approximately 60% of original diameter) for induction of pressure overload-induced left ventricular cardiac hypertrophy. For ensuring successful constriction of the aorta (AC), we recorded pressure gradient across aortic constriction using two-dimensional color Doppler analysis. Twenty rats had a sham operation (SO) as described previously^56,57^. Another 10 rats were considered to control young animals. Echocardiography was performed periodically to assess left ventricular hypertrophy.

Cardiac structure and function in rats were assessed by echocardiography. Systolic intra-ventricular septal thickness (IVSs), diastolic intraventricular septal thickness (IVSd), systolic left ventricular internal dimension (LVIDs), diastolic left ventricular internal dimension (LVIDd), systolic left ventricular posterior wall thickness (LVPWs), diastolic left ventricular posterior wall thickness (LVPWd), left ventricular ejection fraction (LVEF) and left ventricular fractional shortening (LVFS) at the beginning of the experiment, 6 months after aortic constriction or sham operation and 15 months after aortic constriction or sham operation.

### Collection of blood and tissue

Ten rats which underwent aorta constriction and ten sham-operated rats were sacrificed 6 months after the operation, and the remaining 10 rats from both the groups were sacrificed 15 months after the operation. Ten rats of 3 months of age were also sacrificed to assess the cardiac proteome and metabolome in young animals.

Before the sacrifice of animals, body weights, electrocardiogram (ECG), blood pressure (BP) and echocardiogram were recorded. Blood was collected for estimation of levels of serum iron and brain natriuretic peptide (BNP). Heart weights were recorded at the time of sacrifice, and heart weight/body weight ratios were calculated. Heart tissues were excised for histology and molecular analysis.

### Histology

After the sacrifice of all the rats from the three groups, left ventricular tissue samples were collected and fixed in buffered formalin and processed for paraffin embedding. 5 μm thick sections were cut from paraffin-embedded heart tissues. Heart sections were stained with hematoxylin and eosin or Masson’s trichrome. FITC-conjugated wheat germ agglutinin (WGA) stained heart sections were used for measurement of the cross-sectional area of cardiomyocytes. Myocyte hypertrophy and myocardial fibrosis were quantified in heart sections, using software NIS element as described previously^57^.

### Immunohistochemistry analysis

Paraffin-embedded heart tissue sections were deparaffinized and rehydrated. Antigen retrieval was done using citrate buffer (pH 6.8). We followed instructions of the manufacturer as given in Super Sensitive Polymer-HRP, IHC Detection System/DAB kit (QD400-60 K, BioGenex Life Sciences Private Limited, India). After overnight incubation with the ant-ABCB7 antibody at 4 °C, sections were washed with PBS and probed with corresponding secondary antibody. Hematoxylin was used as a nuclear stain for tissue sections. Images were observed under a Nikon microscope (Japan), and histological changes were quantified using software NIS element as described previously^57^.

### Cell culture studies

H9C2, the rat embryo’s heart ventricular cells were obtained from American Type Culture Collection (ATCC). Cells were maintained under culture conditions with 95% DMEM, 5% fetal bovine serum (FBS), and 0.1% penicillin/streptomycin at 37 °C in a 5% CO_2_ incubator. We changed DMEM with Gibco’s Opti-MEM medium in experiments in which treatments with siRNA (100 nM and 200 nM), antimycin (1 μM) and angiotensin II (1 μM) were done. To determine the appropriate dosage for these treatments, pilot experiments were done. Cells were stained with phalloidin and wheat germ agglutinin/WGA (Sigma) for measurement of cell surface area using the software NIS element.

### Biochemical studies

Iron levels in serum and homogenates of heart tissue and H9C2 cells were estimated using iron assay kit (Abcam). Perl’s Prussian blue staining performed using iron staining kit (Abcam). Serum BNP levels were estimated using the BNP-32 rat *in-vitro* ELISA kit (Abcam).

### Proteomic profiling

#### Sample preparation for proteomics

Heart tissues samples (N = 4 from each group) were homogenized with liquid N_2_ using a pestle and mortar. Powdered tissues were lysed using 0.3% Rapigest in 50 mM ammonium bicarbonate buffer at 4 °C. Protein concentration was measured using Bradford assay. 100 μg of protein sample was used for peptide generation by trypsin. Peptide samples were centrifuged at 14000 rpm for 12 minutes, and samples were stored at −20 °C until LC-MS analysis. LC-MS and data analysis were performed as described previously^57^.

#### UPLC-MS analysis

Samples were analyzed using a nano ACQUITY UPLC® System (Waters, UK) coupled to a Quadrupole-Time of Flight (Q/TOF) mass spectrometer (SYNAPT-G2, Waters, UK) controlled by MassLynx4.1 SCN781 software (Waters, UK). Three technical replicate runs were performed for each sample.

#### Data analysis

ProteinLynx Global SERVER™ v2.5.3 (PLGS, Waters, UK) was used for mass spectrometry raw data analysis, which included protein identification as well as relative quantification. *Rattus novergicus* database from NCBI was used for protein annotation. Proteins were identified based on ion match or peptide match. Auto-normalization method was used for normalization. Normalized peak areas of identified peptides were used for label-free quantitative analysis.

Biological Database Network (BioDBnet) tool was used for converting the reference sequence names (Ref Seq) obtained after the PLGS analysis into gene symbols. Database for Annotation, Visualisation, and Integrated Discovery (DAVID) was used for categorizing gene symbols into different pathways. GraphPad Prism (6.0) and MS-Excel 2010 were used for statistical analysis and graphical representations.

### Metabolomics

#### Metabolite extraction from heart tissue

Left ventricular tissues were harvested from 8 animals of each group (N = 5). Samples were washed twice with ice-cold water. Four heart tissues from each group were pooled in two Eppendorf tubes, thus making two replicates per group. Samples were snap-frozen in liquid nitrogen and stored at −80 °C until extraction. An extraction solution containing methanol, acetonitrile, and water (2:2:1 ratio and stored at −20 °C) was used to quench all metabolic reactions simultaneously and to extract intracellular metabolites. Heart tissues (30 mg each) were homogenized in extraction buffer (1 mL) using a mechanical homogenizer and incubated at −80 °C for 10 minutes. Cell suspensions were transferred to 1.5 mL Eppendorf tubes and centrifuged at 14000 rpm at 4 °C for 10 minutes to remove the denatured proteins. Supernatants were collected and stored at −80°C until LC-MS analysis. Each of the 10 samples (2 samples each from 5 groups) were thawed and centrifuged at 14000 rpm for 5 minutes. Supernatants were separated (approximately 200 μL each) and quality control (QC) sample was prepared by pooling 13.3 μL from each sample. A 66.5 μL from sample and QC was dried for analysis in hydrophilic interaction liquid chromatography (HILIC) mode. Dried samples/QCs for HILIC analysis were reconstituted in 80 μL of 80% acetonitrile (ACN), split into two portions and then spiked with reserpine (positive) or taurocholate (negative). 10 μL from each reconstituted sample/QC was injected into the LC-MS system.

#### UHPLC-MS analysis

Separations were performed on a Dionex UltiMate 3000 UHPLC liquid chromatography system (ThermoFisher Scientific, San Jose, CA, USA). Chromatography was carried out using a Phenomenex Luna column (5 μm pore size and 150 mm × 4.6 mm HILIC dimensions). Mobile phase A consisted of 5 mM ammonium carbonate dissolved in water and 5 mM ammonium carbonate dissolved in acetonitrile was used as mobile phase B. Gradient program started with 100% mobile phase B and then linearly decreased to 90% at 15 minutes, to 80% after 10 minutes, to 75% at 30 minutes and to 20% at 35 minutes and finally held for 5 minutes. Mobile phase B was reduced to 0%/100% mobile phase A for 5 minutes and then held for 10 minutes. The total run time was 55 minutes, with a flow rate of 400 μL/min. Samples were maintained at 4 °C during the procedure, and the injection volume was 10 μL per sample. MS analysis was performed using a Q-Exactive Orbitrap (Thermo Fisher Scientific, San Jose, CA, USA) with a heated electron spray ionisation-II (HESI-II) probe. MS parameters used were as follows: resolution 140000; scan range (m/z) 70–1050. Immediately before analysis of each batch, mass calibration was performed for each polarity.

#### Data analysis

Raw files were imported into the software Progenesis QI (Nonlinear dynamics, Waters) for extraction of metabolite features and generation of peak list. Progenesis QI was also used for searching Human Metabolite Database (HMDB) for possible identification. The metabolite features were identified based on theoretical fragmentation score, mass error, molecular formula, and isotope similarity. Annotations with maximum score were accepted for further analysis. Data were filtered for removal of features using conditions such as coefficient of variation (CV) of QCs > 20 and presence in less than 70% of QC samples (Supplementary Fig. 3). Filtered peak list was imported into MetaboAnalyst 3.0 for principal component analysis (PCA) and to check the quality of data. Pathway analysis was done for differentially expressed metabolites (*P* < 0.05 and fold change > 2) using integrated molecular pathway-level analysis (IMPaLA) software. The features uniquely identified in a group (labeled as infinity in Progenesis QI result table) were given a value of 10 (higher than the maximum value of any feature from data). Fold change values were converted to log 10 scales for a graphical representation (Supplementary Figs S1 and S2).

### Isolation of mitochondria from rat heart and H9C2 cells

We followed the protocol described by Frezza *et al*., for the isolation of mitochondria^58^. Immediately after the sacrifice of the animals, 25 mg of left ventricles were excised from 4 rats of each experimental group. After mincing, the ventricular tissue bits were washed twice with ice-cold PBS-EDTA and incubated in ice-cold PBS-EDTA (10 mM) and 0.01% trypsin for 30 minutes. Tissue bits were then centrifuged at 200 g for 5 minutes at 4 °C. The pellets were resuspended in mitochondrial isolation buffer 1 (IBm1) and homogenized using a Teflon pestle at 1600 rpm at 4 °C (the minced muscle was stroked ten times). Homogenates were then centrifuged at 700 g, 4 °C for 10 minutes. Pellets were resuspended in approximately 5 ml of ice-cold mitochondrial isolation buffer 2 (IBm2 buffer) and centrifuged at 8000 g at 4 °C for 10 minutes. Pellets were resuspended in 200 μL of IBm2 buffer. Supernatants were collected in fresh tubes for analysis of cytoplasmic proteins. Mitochondrial suspensions were analyzed for protein concentration using the Bradford assay. The average protein concentration from the mitochondrial suspension was ~30 mg/mL. The same protocol was used for isolation of mitochondria from H9C2 cells.

### Cloning of ABCB7gene

RNA was isolated from H9C2 cells using RNeasy^®^ Micro Kit (Qiagen). Total RNA was converted into cDNA using High Capacity cDNA Reverse Transcription Kits (Applied Biosystems). Gene end-specific primers were designed, and restriction sites for EcoRI and HindIII were added at the 5′-flanking ends of the forward and reverse primers respectively. Full gene was amplified by PCR using primers, the PCR product was run in gel and eluted using Gel Elution Kit (Qiagen). For cloning, 1 µg each of the PCR product and circular pCMV-Tag2a vector was kept for digestion under optimal conditions recommended for the restriction enzymes used. Digested products were run in an electrophoresis gel, eluted and kept for ligation overnight at 4°C. Clones were transformed into *E.coli* DH5α strain and plated over LB agar kanamycin containing plates. Colony PCR was performed to identify the clone and identity of the cloned gene was confirmed by DNA sequencing (3730 DNA analyzer).

### siRNA and flag-ABCB7 transfection

H9C2 cells were transfected using siRNA (Sense 5′-AUCCAUGUGGCAUUCGAAG-3′ and anti-sense 5′-CUUCGAAUGCCACAUGGAU-3′) targeting the rat ABCB7 gene or scrambled siRNA (negative control). Manufacturer’s instructions were followed. Briefly, the cells were seeded in a six-well plate and at approximately 70% confluency, transfected with either siRNA (100 nM and 200 nM) or flag-ABCB7 (full-length, 2.5 μg/μL; plasmid to Lipofectamine ratio of 1:2) in Opti MEM medium (serum and antibiotic-free) containing 5 μL of Lipofectamine (Invitrogen). After 6 hours of incubation at 37 °C in a CO_2_ incubator, the medium was replaced with normal DMEM supplemented with FBS. After 24 hours, cells were harvested for gene and protein expression studies.

### TMRM (Tetramethylrhodamine, methyl ester) staining

H9C2 cells were seeded in a 6 well plate and allowed to become confluent up to 70%. Fresh medium was replaced in all the wells after 6 hours of transfection with either siABCB7. TMRM staining solution (100 nM) was added to each well, and cells were incubated at 37 °C for 30 minutes. Cells were trypsinized and analyzed under a fluorescence-activated cell sorter (FACS) (Becton Dickinson) using FACS Diva v8.0 software at 579 nm wavelength. For immunofluorescence analysis, cells were observed immediately after incubation with TMRM under a confocal microscope (Leica SP2 Laser Scanning Spectral Confocal system) at 568 nm wavelength. Images were analyzed and quantified using software NIS-Elements.

### Measurement of ROS production

#### Amplex Red (10-acetyl-3,7-dihydroxyphenoxazine) hydrogen peroxide assay

Mitochondrial and cytoplasmic fractions isolated from heart tissues and H9C2 cells were analyzed for hydrogen peroxide levels. We followed the manufacturer’s instructions (Amplex Red hydrogen peroxide/peroxidase assay kit, ThermoFisher Scientific) for measurement of hydrogen peroxide. Briefly, 50 μL of the sample (test, a positive control (10 μM H_2_O_2_) and negative control (1X reaction buffer) per well was loaded into a 96 well microplate. 50 μL of Amplex Red reagent/HRP working solution (100 μM) was added to each microplate well. The microplate was incubated for 30 minutes at room temperature (under dark), and the absorbance was measured at 560 nm in a microplate reader (BioRad). H_2_O_2_ level was quantified from these readings and corrected for the background absorbance by subtracting the negative control value from the test value.

#### MitoSOX Red assay

This assay was used for measurement of superoxide levels in angiotensin II-stimulated H9C2 cells after silencing ABCB7. Cells were seeded on a 96 well microplate and kept at 37 °C in a CO_2_ incubator. After transfection or drug treatment, cells were loaded with MitoSOX Red (5 mM) for 15 minutes. Cells were observed using confocal microscopy and FACS for image analysis.

### Immunofluorescence

H9C2 cells were cultured in 24 well plates for 24 hours at 37 °C in a CO_2_ incubator. After 70% confluence of cells, the medium was replaced, and drugs or siABCB7 or flag-ABCB7 was added to each well. After appropriate incubation (according to manufacturer’s instructions or protocol described earlier), the medium was removed from the cells and cells were washed with PBS (pH 7.4). Cells were fixed with 4% paraformaldehyde and permeabilized with Triton-X100 (0.1%). Cells were incubated with 3% BSA (blocking solution) at room temperature for 30 minutes and subsequently incubated at 4 °C for overnight with primary antibodies. Cells were then washed with PBS and incubated with fluorescent-labeled secondary antibodies for one hour at room temperature (this step was skipped if the fluorescent-labeled primary antibody was used). Cells were washed with PBS and stained with 4′, 6-diamidino-2-phenylindole (DAPI), a nuclear dye. After staining, cells were observed under a confocal microscope (Leica SP2 Laser Scanning Spectral Confocal system) at different wavelengths and images were analyzed using software NIS-Elements.

### Western blot

Snap-frozen heart tissue collected after the sacrifice of rats were minced, ground and homogenized with Teflon pestle or pestle and mortar in liquid N_2_. The homogenized tissue samples were extracted in a Radioimmunoprecipitation assay buffer (RIPA buffer) with a protease inhibitor mixture (Roche). Mitochondrial and cytoplasmic protein fractions were obtained as described previously.

H9C2 cells were harvested after transfection or drug treatment, washed twice with ice-cold PBS-EDTA and extracted in RIPA buffer as mentioned above. Protein concentration in each sample was determined using the Bradford assay. 20 μg of proteins were loaded on different percentages of SDS-PAGE gel and transferred to Polyvinylidene fluoride (PVDF) membranes. The membrane was probed with several primary antibodies listed in Supplementary Table S7. Immunoblots were quantified as described previously^57^.

### qRT-PCR

Total RNA was isolated from snap-frozen heart tissues or H9C2 cells using RNeasy Mini Kit (Qiagen). RNA (2 μg) was reverse transcribed to complementary DNA using reverse transcriptase (NEB) and quantitative real-time PCR was performed with SYBR green (Applied Biosystems). Primers efficiency was determined before gene expression analysis and specificity of primers (single product amplification) were assessed by melting curve analysis. The mRNA expression was quantified by using 2^ΔΔ C^_T_ method, normalized with GAPDH, and data were represented as relative fold change of target gene for test compared with control samples. Primer sequences used are listed in Supplementary Table S8.

### Co-Immunoprecipitation

H9C2 cells were harvested, suspended and sonicated (30 seconds on/off cycle) in IP buffer (20 mM Tris-HCL, 1 mM EDTA, 150 mM NaCl and 0.5% NP-40) and mixed with a protease inhibitor (Roche) immediately before use. Cells were washed using ice-cold PBS and centrifuged at 4 °C for 20 minutes. Cell lysates were collected in Eppendorf tubes. Cell lysates (~1 mg protein) was immunoprecipitated with either antibody (2–3 μg) or protein A/G bound to agarose beads at 4 °C and incubated overnight. Immunoprecipitated complex was washed five to six times with IP buffer and eluted in 2X Laemmali sample buffer. 100 μg samples were loaded in SDS-PAGE and immunoblotted as described earlier.

### Statistical analysis

All of the data are expressed as mean ± SEM. Statistical analysis of data was performed using the software GraphPad Prism 6.0. One-way ANOVA, two-way ANOVA followed by multiple comparisons with Tukey’s or Bonferroni test wherever applicable. *P* < *0.05* was considered as statistically significant. The volcano plots were constructed using R 3.2.5 software. Peak intensity table of metabolites was used for the volcano plot construction. Fold change values obtained from PLGS software was used for proteomics data analysis. A significant probability of upregulation value was coded as a *p* < 0.01 for proteins represented in volcano plots^59^.

## Supplementary information

Dataset 1

Supplementary Information
