# Supplementary material for: Chronic Pressure Overload Results in Deficiency of Mitochondrial Membrane Transporter ABCB7 Which Contributes to Iron Overload, Mitochondrial Dysfunction, Metabolic Shift and Worsens Cardiac Function
Source: Sci Rep. 2019 Sep 11;9:13170. doi: 10.1038/s41598-019-49666-0 (PMC6739357; doi:10.1038/s41598-019-49666-0)
Supplement: Supplementary file 2 — Supplementary Information [file 41598_2019_49666_MOESM2_ESM.pdf]

Chronic Pressure Overload Results in Deficiency of Mitochondrial Membrane Transporter ABCB7 Which Contributes to Iron Overload, Mitochondrial Dysfunction, Metabolic Shift and Worsens Cardiac Function

Vikas Kumar[1, 3], Aneesh Kumar A[1, 3], Rahul Sanawar[2, 3], Abdul Jaleel[1,3], TR Santhosh Kumar[1, 2, 3*], CC Kartha[1*].

[1]Cardiovascular Diseases and Diabetes Biology, Rajiv Gandhi Centre for Biotechnology (RGCB), Trivandrum, Kerala, India

[2]Cancer Research Program, Rajiv Gandhi Centre for Biotechnology (RGCB), Trivandrum, Kerala, India

[3]Graduate studies, Manipal Academy of Higher Education (MAHE), Manipal, Karnataka, India

Address correspondence to:

Dr. CC Kartha
Cardiovascular Diseases and Diabetes Biology
Rajiv Gandhi Centre for Biotechnology
Poojappura, Thycaud Post
Trivandrum-695014
Kerala, India
Tel: +91-0471 2529484
Fax: +91 0471 2529505
cckartha@gmail.com
drkartha.cc@kimsglobal.com

Dr. TR Santhosh Kumar
Cardiovascular Diseases and Diabetes Biology
Rajiv Gandhi Centre for Biotechnology
Poojappura, Thycaud Post
Trivandrum-695014
Kerala, India
Tel: +91-0471 2529484
Fax: +91 0471 2348096
trsanthosh@rgcb.res.in

Supplementary Table S1. Fatty acid/lipid metabolites in heart tissues of rats which underwent sham operation (sacrificed at 6 months after sham operation) when compared with 3 months old rats.

Compound ID	Description	Adducts	Formula	m/z	**log10** (3M Vs 6M SO)	Mode
HMDB00122	D-Glucose	M-H	C6H12O6	179.05	1.35	HILIC_Neg
HMDB01553	2-Oxo-4-methylthiobutanoic acid	M+FA-H	C5H8O3S	193.01	1	HILIC_Neg
HMDB00535	Caproic acid	M+H-2H2O	C6H12O2	81.07	0.76	HILIC_pos
HMDB00172	L-Isoleucine	M+H-H2O	C6H13NO2	114.09	0.39	HILIC_pos
HMDB03556	Chitobiose	M+H-H2O, M+H	C15H26N2O12	427.15	-0.50	HILIC_pos
HMDB00732	Hydroxykynurenine	M+H-2H2O	C10H12N2O4	189.06	-0.76	HILIC_pos
HMDB01229	Dopaquinone	M+H	C9H9NO4	196.06	-1	HILIC_pos
HMDB00064	Creatine	M+H-H2O	C4H9N3O2	114.06	-1.12	HILIC_pos

Supplementary Table S2. Fatty acid/lipid metabolites in heart tissue of rats which underwent sham operation (sacrificed at 15 months after sham operation) when compared with 3 months old rats.

Compound ID	Description	Adducts	Formula	m/z	**log10** (3M Vs 15M SO)	Mode
HMDB00122	D-Glucose	M-H	C6H12O6	179.05	1.12	HILIC_Neg
HMDB00262	Thymine	M+NH4	C5H6N2O2	144.07	1	HILIC_pos
HMDB07850	LPA(0:0/18:0)	M+K-2H	C21H43O7P	475.22	0.93	HILIC_Neg
HMDB01229	Dopaquinone	M+H	C9H9NO4	196.06	0.90	HILIC_pos
HMDB03556	Chitobiose	M+H	C15H26N2O12	427.15	0.75	HILIC_pos
HMDB07955	PC(15:0/22:4(7Z,10Z,13Z,16Z))	M+H-2H2O	C45H82NO8P	760.56	0.55	HILIC_pos
HMDB09264	PE(20:1(11Z)/20:2(11Z,14Z))	M+FA-H	C45H84NO8P	842.59	0.46	HILIC_Neg
HMDB09000	PE(18:0/20:2(11Z,14Z))	M+FA-H	C43H82NO8P	816.57	0.40	HILIC_Neg
HMDB02183	Docosahexaenoic acid	M-H	C22H32O2	327.23	0.39	HILIC_Neg
HMDB00168	L-Asparagine	M+H-2H2O	C4H8N2O3	97.04	0.31	HILIC_pos

HMDB01548	D-Ribose 5-phosphate	M-H	C5H11O8P	229.01	-0.30	HILIC_Neg
HMDB00535	Caproic acid	M+H	C6H12O2	117.09	-0.40	HILIC_pos
HMDB00904	Citrulline	M-H	C6H13N3O3	174.08	-0.45	HILIC_Neg
HMDB14672	Cyclophosphamide	M+H, M+NH4	C7H15Cl2N2O2P	278.05	-0.47	HILIC_pos
HMDB01014	4-Imidazolone-5-propionic acid	M+H-H2O	C6H8N2O3	139.05	-0.48	HILIC_pos
HMDB10382	LysoPC(16:0)	M+FA-H	C24H50NO7P	540.33	-0.61	HILIC_Neg
HMDB00222	L-Palmitoylcarnitine	M+H	C23H45NO4	400.34	-0.64	HILIC_pos
HMDB02815	LysoPC(18:1(9Z))	M+FA-H	C26H52NO7P	566.34	-1	HILIC_Neg
HMDB09103	PE(18:2(9Z,12Z)/20:4(8Z,11Z,14Z,17Z))	M-H	C43H74NO8P	762.50	-1	HILIC_Neg
HMDB09411	PE(20:4(5Z,8Z,11Z,14Z)/P-16:0)	M-H	C41H74NO7P	722.51	-1	HILIC_Neg
HMDB09602	PE(22:4(7Z,10Z,13Z,16Z)/22:4(7Z,10Z,13Z,16Z))	M+FA-H	C49H82NO8P	888.57	-1	HILIC_Neg
HMDB11111	Malonic semialdehyde	2M-H	C3H4O3	175.02	-1	HILIC_Neg
HMDB00056	Beta-Alanine	M+FA-H	C3H7NO2	134.04	-1.23	HILIC_Neg
HMDB00085	Deoxyguanosine	M-H, M+Cl, M+FA-H	C10H13N5O4	312.09	-1.44	HILIC_Neg

Supplementary Table S3. Metabolic profile from heart tissues of rats which underwent aortic constriction (sacrificed at 6 months after aortic constriction) when compared with 3 months old rats.

**Compound ID**	**Description**	**Adducts**	**Formula**	**m/z**	**log10** (3M Vs 6M AC)	**Mode**
HMDB00207	Oleic acid	M-H	C18H34O2	281.24	0.91	HILIC_Neg
HMDB00208	Oxoglutaric acid	M+FA-H	C5H6O5	191.01	4.54	HILIC_Neg
HMDB00220	Palmitic acid	M-H	C16H32O2	255.23	0.70	HILIC_Neg
HMDB00262	Thymine	M+NH4	C5H6N2O2	144.07	1	HILIC_pos
HMDB00573	Elaidic acid	M-H	C18H34O2	281.24	0.91	HILIC_Neg
HMDB00673	Linoleic acid	M-H	C18H32O2	279.23	1.13	HILIC_Neg
HMDB00827	Stearic acid	M-H, M+FA-H	C18H36O2	283.26	0.80	HILIC_Neg
HMDB01043	Arachidonic acid	M-H, 2M-H	C20H32O2	303.23	1.57	HILIC_Neg
HMDB01229	Dopaquinone	M+H	C9H9NO4	196.06	0.93	HILIC_pos

HMDB01553	2-Oxo-4-methylthiobutanoic acid	M+FA-H	C5H8O3S	193.01	1	HILIC_Neg
HMDB02183	Docosahexaenoic acid	M-H	C22H32O2	327.23	1.91	HILIC_Neg
HMDB02226	Adrenic acid	M-H	C22H36O2	331.26	0.79	HILIC_Neg
HMDB02815	LysoPC(18:1(9Z))	M+FA-H	C26H52NO7P	566.34	2.09	HILIC_Neg
HMDB02925	8,11,14-Eicosatrienoic acid	M-H	C20H34O2	305.24	1.13	HILIC_Neg
HMDB03556	Chitobiose	M+H	C15H26N2O12	427.15	0.59	HILIC_pos
HMDB07850	LPA(18:0/0:0)	M+FA-H	C21H43O7P	483.27	1	HILIC_Neg
HMDB07852	LPA(0:0/18:2(9Z,12Z)	M-H	C21H37O6P	415.22	1	HILIC_Neg
HMDB07854	LPA(0:0/18:0)	M+FA-H	C21H43O7P	483.27	1	HILIC_Neg
HMDB07856	CPA(16:0/0:0)	M-H	C19H37O6P	391.22	1	HILIC_Neg
HMDB09411	PE(20:4(5Z,8Z,11Z,14Z)/P-16:0)	M-H	C41H74NO7P	722.51	0.55	HILIC_Neg
HMDB10382	LysoPC(16:0)	M+FA-H	C24H50NO7P	540.33	0.87	HILIC_Neg
HMDB10385	LysoPC(18:1(11Z))	M+FA-H	C26H52NO7P	566.34	2.09	HILIC_Neg
HMDB10386	LysoPC(18:2(9Z,12Z))	M+FA-H	C26H50NO7P	564.33	1	HILIC_Neg
HMDB12108	LysoPC(17:0)	M+FA-H	C25H52NO7P	554.34	0.97	HILIC_Neg
HMDB00056	Beta-Alanine	M+FA-H	C3H7NO2	134.04	-1.06	HILIC_Neg
HMDB00085	Deoxyguanosine	M-H, M+Cl, M+FA-H	C10H13N5O4	312.09	-1.30	HILIC_Neg
HMDB00115	Glycolic acid	2M-H	C2H4O3	151.02	-0.86	HILIC_Neg
HMDB00201	L-Acetylcarnitine	M+H	C9H17NO4	204.12	-1.28	HILIC_pos
HMDB00660	D-Fructose	M-H	C6H12O6	179.05	-1	HILIC_Neg
HMDB01343	Mevalonic acid-5P	M+FA-H	C6H13O7P	273.03	-1	HILIC_Neg
HMDB01406	Niacinamide	M+H	C6H6N2O	123.05	-0.98	HILIC_pos
HMDB01548	D-Ribose 5-phosphate	M-H	C5H11O8P	229.01	-1	HILIC_Neg
HMDB11111	Malonic semialdehyde	2M-H	C3H4O3	175.02	-1	HILIC_Neg
HMDB14672	Cyclophosphamide	M+H, M+NH4	C7H15Cl2N2O2P	278.05	-2.51	HILIC_pos
HMDB14732	Amiloride	M+Na	C6H8ClN7O	252.03	-1.88	HILIC_pos

Supplementary Table S4. Metabolic profile from heart tissues of rats which underwent aortic constriction (sacrificed at 15 months after aortic constriction) when compared with 3 months old rats.

**Compound ID**	**Description**	**Adducts**	**Formula**	**m/z**	**log10** (3M Vs 15M AC)	**Mode**
HMDB00208	Oxoglutaric acid	M+FA-H	C5H6O5	191.01	4.50	HILIC_Neg
HMDB02183	Docosahexaenoic acid	M-H	C22H32O2	327.23	2.02	HILIC_Neg
HMDB02815	LysoPC(18:1(9Z)),LysoPC(18:1(11Z))	M+FA-H	C26H52NO7P	566.34	1.81	HILIC_Neg
HMDB10385	LysoPC(18:1(11Z))	M+FA-H	C26H52NO7P	566.34	1.81	HILIC_Neg
HMDB01043	Arachidonic acid	M-H, 2M-H	C20H32O2	303.23	1.52	HILIC_Neg
HMDB09602	PE(22:4(7Z,10Z,13Z,16Z)/22:4(7Z,10Z,13Z,16Z))	M+FA-H	C49H82NO8P	888.57	1.10	HILIC_Neg
HMDB01229	Dopaquinone	M+H	C9H9NO4	196.06	1.04	HILIC_pos
HMDB00673	Linoleic acid	M-H	C18H32O2	279.23	1.01	HILIC_Neg
HMDB02226	Adrenic acid	M-H	C22H36O2	331.26	1.01	HILIC_Neg
HMDB00848	Stearoylcarnitine	M+K-2H	C25H49NO4	464.31	1	HILIC_Neg
HMDB01553	2-Oxo-4-methylthiobutanoic acid	M+FA-H	C5H8O3S	193.01	1	HILIC_Neg
HMDB07852	LPA(0:0/18:2(9Z,12Z)	M-H	C21H37O6P	415.22	1	HILIC_Neg
HMDB07856	LPA(18:2(9Z,12Z)/0:0)	M-H	C21H37O6P	415.22	1	HILIC_Neg
HMDB10386	LysoPC(18:2(9Z,12Z))	M+FA-H	C26H50NO7P	564.33	1	HILIC_Neg
HMDB00904	Citrulline	M-H	C6H13N3O3	174.08	1	HILIC_Neg
HMDB01343	Mevalonic acid-5P	M+FA-H	C6H13O7P	273.03	1	HILIC_Neg
HMDB01548	D-Ribose 5-phosphate	M-H	C5H11O8P	229.01	1	HILIC_Neg
HMDB11111	Malonic semialdehyde	2M-H	C3H4O3	175.02	1	HILIC_Neg
HMDB07850	LPA(0:0/18:0)	M+K-2H	C21H43O7P	475.22	0.95	HILIC_Neg
HMDB07854	LPA(18:0/0:0)	M+K-2H	C21H43O7P	475.22	0.95	HILIC_Neg
HMDB07854	LPA(18:0/0:0)	M+K-2H	C21H43O7P	475.22	0.95	HILIC_Neg
HMDB12108	LysoPC(17:0)	M+FA-H	C25H52NO7P	554.34	0.89	HILIC_Neg
HMDB00207	Oleic acid	M-H	C18H34O2	281.24	0.85	HILIC_Neg
HMDB00573	Elaidic acid	M-H	C18H34O2	281.24	0.85	HILIC_Neg
HMDB00827	Stearic acid	M-H, M+FA-H	C18H36O2	283.26	0.84	HILIC_Neg

HMDB02925	8,11,14-Eicosatrienoic acid	M-H	C20H34O2	305.24	0.83	HILIC_Neg
HMDB10382	LysoPC(16:0)	M+FA-H	C24H50NO7P	540.33	0.71	HILIC_Neg
HMDB00220	Palmitic acid	M-H	C16H32O2	255.23	0.64	HILIC_Neg
HMDB09028	PE(18:1(11Z)/18:3(6Z,9Z,12Z)	M-H	C41H74NO8P	738.51	-0.31	HILIC_Neg
HMDB09029	PE(18:1(11Z)/18:3(9Z,12Z,15Z)	M-H	C41H74NO8P	738.51	-0.31	HILIC_Neg
HMDB09061	PE(18:1(9Z)/18:3(6Z,9Z,12Z)	M-H	C41H74NO8P	738.51	-0.31	HILIC_Neg
HMDB09062	PE(18:1(9Z)/18:3(9Z,12Z,15Z)	M-H	C41H74NO8P	738.51	-0.31	HILIC_Neg
HMDB07861	PA(18:0/18:2(9Z,12Z))	M+FA-H	C39H73O8P	745.50	-0.47	HILIC_Neg
HMDB09000	PE(18:0/20:2(11Z,14Z))	M+FA-H	C43H82NO8P	816.57	-0.47	HILIC_Neg
HMDB09288	PE(20:2(11Z,14Z)/18:0)	M+FA-H	C43H82NO8P	816.57	-0.47	HILIC_Neg
HMDB00296	Uridine	M-H, M+Cl	C9H12N2O6	243.06	-0.53	HILIC_Neg
HMDB00115	Glycolic acid	2M-H	C2H4O3	151.02	-0.71	HILIC_Neg
HMDB01406	Niacinamide	M+H	C6H6N2O	123.05	-0.77	HILIC_pos
HMDB00085	Deoxyguanosine	M-H, M+Cl, M+FA-H	C10H13N5O4	312.09	-1.13	HILIC_Neg
HMDB00201	L-Acetylcarnitine	M+H	C9H17NO4	204.12	-1.19	HILIC_pos
HMDB00247	Sorbitol	M-H, M+Cl	C6H14O6	181.07	-1.37	HILIC_Neg
HMDB14672	Cyclophosphamide	M+H, M+NH4	C7H15Cl2N2O2P	278.05	-2.66	HILIC_pos

Supplementary Table S5. Metabolic profile from heart tissues of rats which underwent aortic constriction or sham operation (sacrificed at 6 months after aortic constriction) when compared with 3 months old rats.

**Compound ID**	**Description**	**Adducts**	**Formula**	**m/z**	**log10** (6M AC Vs 6M SO)	**Mode**
HMDB00064	Creatine	M+NH4	C5H6N2O2	144.07	1	HILIC_pos
HMDB00207	Oleic acid	M-H	C18H34O2	281.24	0.86	HILIC_Neg
HMDB00208	Oxoglutaric acid	M+FA-H	C5H6O5	191.01	4.49	HILIC_Neg
HMDB00220	Palmitic acid	M-H	C16H32O2	255.23	0.64	HILIC_Neg
HMDB00262	Thymine	M+NH4	C5H6N2O2	144.07	1	HILIC_pos

HMDB00573	Elaidic acid	M-H	C18H34O2	281.24	0.86	HILIC_Neg
HMDB00673	Linoleic acid	M-H	C18H32O2	279.23	1.11	HILIC_Neg
HMDB00827	Stearic acid	M-H	C18H36O2	283.26	0.77	HILIC_Neg
HMDB00848	Stearoylcarnitine	M+K-2H	C25H49NO4	464.31	1	HILIC_Neg
HMDB01043	Arachidonic acid	M-H	C20H32O2	303.23	1.52	HILIC_Neg
HMDB01229	Dopaquinone	M+H	C9H9NO4	196.06	1	HILIC_pos
HMDB01553	2-Oxo-4-methylthiobutanoic acid	M+FA-H	C5H8O3S	193.01	2.01	HILIC_Neg
HMDB02183	Docosahexaenoic acid	M-H	C22H32O2	327.23	1.78	HILIC_Neg
HMDB02226	Adrenic acid	M-H	C22H36O2	331.26	0.77	HILIC_Neg
HMDB02815	LysoPC(18:1(9Z))	M+FA-H	C26H52NO7P	566.34	1.99	HILIC_Neg
HMDB02925	8,11,14-Eicosatrienoic acid	M-H	C20H34O2	305.24	1.09	HILIC_Neg
HMDB03556	Chitobiose	M+H	C15H26N2O12	427.15	1.06	HILIC_pos
HMDB07850	LPA(0:0/18:0)	M+FA-H	C21H43O7P	483.27	1	HILIC_Neg
HMDB07852	LPA(0:0/18:2(9Z,12Z))	M-H	C21H37O6P	415.22	1	HILIC_Neg
HMDB07854	LPA(18:0/0:0)	M+FA-H	C21H43O7P	483.27	1	HILIC_Neg
HMDB10382	LysoPC(16:0)	M+FA-H	C24H50NO7P	540.33	0.96	HILIC_Neg
HMDB10385	LysoPC(18:1(11Z))	M+FA-H	C26H52NO7P	566.34	1.99	HILIC_Neg
HMDB10386	LysoPC(18:2(9Z,12Z))	M+FA-H	C26H50NO7P	564.33	1	HILIC_Neg
HMDB10395	LysoPC(20:4(5Z,8Z,11Z,14Z))	M+FA-H	C28H50NO7P	588.33	1	HILIC_Neg
HMDB11631	L-3-Hydroxykynurenine	M+H-2H2O	C10H12N2O4	189.06	0.53	HILIC_pos
HMDB12108	LysoPC(17:0)	M+FA-H	C25H52NO7P	554.34	1.02	HILIC_Neg
HMDB40733	Phenyl acetate	M+H	C8H8O2	137.05	0.81	HILIC_pos
HMDB00056	Beta-Alanine	M+FA-H	C3H7NO2	134.04	-0.92	HILIC_Neg
HMDB00085	Deoxyguanosine	M-H, M+Cl, M+FA-H	C10H13N5O4	312.09	-1.30	HILIC_Neg
HMDB00115	Glycolic acid	2M-H	C2H4O3	151.02	-0.83	HILIC_Neg
HMDB00122	D-Glucose	M-H	C6H12O6	179.05	-1.53	HILIC_Neg
HMDB00143	D-Galactose	M-H	C6H12O6	179.05	-1.53	HILIC_Neg
HMDB00201	L-Acetylcarnitine	M+H	C9H17NO4	204.12	-1.30	HILIC_pos

HMDB00210	Pantothenic acid	M+H	C9H17NO5	220.11	-0.59	HILIC_pos
HMDB00516	Beta-D-Glucose	M-H	C6H12O6	179.05	-1.53	HILIC_Neg
HMDB00535	Caproic acid	M+H-2H2O	C6H12O2	81.07	-0.61	HILIC_pos
HMDB00944	Behenic acid	M+NH4	C22H44O2	358.36	-1.02	HILIC_pos
HMDB01343	Mevalonic acid-5P	M+FA-H	C6H13O7P	273.03	-1	HILIC_Neg
HMDB01406	Niacinamide	M+H	C6H6N2O	123.05	-1.02	HILIC_pos
HMDB01548	D-Ribose 5-phosphate	M-H	C5H11O8P	229.01	-1	HILIC_Neg
HMDB11111	Malonic semialdehyde	2M-H	C3H4O3	175.02	-1	HILIC_Neg
HMDB14672	Cyclophosphamide	M+H, M+NH4	C7H15Cl2N2O2P	278.05	-2.55	HILIC_pos
HMDB14732	Amiloride	M+Na	C6H8ClN7O	252.03	-1.61	HILIC_pos

Supplementary Table S6. Metabolic profile from heart tissues of rats which underwent aortic constriction or sham operation (sacrificed at 15 months after aortic constriction) when compared with 3 months old rats.

**Compound ID**	**Description**	**Adducts**	**Formula**	**m/z**	**log10** (15M AC Vs 15M SO)	**Mode**
HMDB00207	Oleic acid	M-H	C18H34O2	281.24	0.69	HILIC_Neg
HMDB00220	Palmitic acid	M-H	C16H32O2	255.23	0.40	HILIC_Neg
HMDB00573	Elaidic acid	M-H	C18H34O2	281.24	0.69	HILIC_Neg
HMDB00673	Linoleic acid	M-H	C18H32O2	279.23	0.84	HILIC_Neg
HMDB00827	Stearic acid	M-H, M+FA-H	C18H36O2	283.26	0.57	HILIC_Neg
HMDB01043	Arachidonic acid	M-H, 2M-H	C20H32O2	303.23	1.36	HILIC_Neg
HMDB02183	Docosahexaenoic acid	M-H	C22H32O2	327.23	1.62	HILIC_Neg
HMDB02226	Adrenic acid	M-H	C22H36O2	331.26	0.89	HILIC_Neg
HMDB02815	LysoPC(18:1(9Z))	M+FA-H	C26H52NO7P	566.34	1	HILIC_Neg
HMDB02925	8,11,14-Eicosatrienoic acid	M-H	C20H34O2	305.24	0.72	HILIC_Neg
HMDB07850	LPA(0:0/18:0)	M+FA-H	C21H43O7P	483.27	1	HILIC_Neg
HMDB07852	LPA(0:0/18:2(9Z,12Z))	M-H	C21H37O6P	415.22	1	HILIC_Neg
HMDB07854	LPA(18:0/0:0)	M+FA-H	C21H43O7P	483.27	1	HILIC_Neg

HMDB07856	LPA(18:2(9Z, 12Z)/0:0)	M-H	C21H37O6P	415.22	1	HILIC_Neg
HMDB09103	PE(18:2(9Z,12Z)/20:4(8Z,11Z,14Z,17Z))	M-H	C43H74NO8P	762.50	1	HILIC_Neg
HMDB09411	PE(20:4(5Z,8Z,11Z,14Z)/P-16:0)	M-H	C41H74NO7P	722.51	1	HILIC_Neg
HMDB09423	PE(20:4(8Z,11Z,14Z,17Z)/18:2(9Z,12Z))	M-H	C43H74NO8P	762.50	1	HILIC_Neg
HMDB09602	PE(22:4(7Z,10Z,13Z,16Z)/22:4(7Z,10Z,13Z,16Z))	M+FA-H	C49H82NO8P	888.57	1	HILIC_Neg
HMDB10382	LysoPC(16:0)	M+FA-H	C24H50NO7P	540.33	1.32	HILIC_Neg
HMDB10385	LysoPC(18:1(11Z))	M+FA-H	C26H52NO7P	566.34	1	HILIC_Neg
HMDB10386	LysoPC(18:2(9Z,12Z))	M+FA-H	C26H50NO7P	564.33	1	HILIC_Neg
HMDB10395	LysoPC(20:4(5Z,8Z,11Z,14Z))	M+FA-H	C28H50NO7P	588.33	1	HILIC_Neg
HMDB12108	LysoPC(17:0)	M+FA-H	C25H52NO7P	554.34	1.00	HILIC_Neg
HMDB00115	Glycolic acid	2M-H	C2H4O3	151.02	-0.58	HILIC_Neg
HMDB00122	D-Glucose	M-H	C6H12O6	179.05	-1.23	HILIC_Neg
HMDB00143	D-Galactose	M-H	C6H12O6	179.05	-1.23	HILIC_Neg
HMDB00169	D-Mannose	M-H	C6H12O6	179.05	-1.23	HILIC_Neg
HMDB00201	L-Acetylcarnitine	M+H	C9H17NO4	204.12	-0.94	HILIC_pos
HMDB00247	Sorbitol	M-H, M+Cl	C6H14O6	181.07	-1.15	HILIC_Neg
HMDB00516	Beta-D-Glucose	M-H	C6H12O6	179.05		HILIC_Neg
HMDB00904	Citrulline	M-H	C6H13N3O3	174.08	-1	HILIC_Neg
HMDB01343	Mevalonic acid-5P	M+FA-H	C6H13O7P	273.03	-1	HILIC_Neg
HMDB01406	Niacinamide	M+H	C6H6N2O	123.05	-0.50	HILIC_pos
HMDB01548	D-Ribose 5-phosphate	M-H	C5H11O8P	229.01	-1	HILIC_Neg
HMDB07861	PA(18:0/18:2(9Z,12Z))	M+FA-H	C39H73O8P	745.50	-0.47	HILIC_Neg
HMDB07955	PC(15:0/22:4(7Z,10Z,13Z,16Z))	M+H-2H2O	C45H82NO8P	760.56	-0.78	HILIC_pos

HMDB09000	PE(18:0/20:2(11Z,14Z))	M+FA-H	C43H82NO8P	816.57	-0.88	HILIC_Neg
HMDB09264	PE(20:1(11Z)/20:2(11Z,14Z))	M+FA-H	C45H84NO8P	842.59	-0.47	HILIC_Neg
HMDB09288	PE(20:2(11Z,14Z)/18:0)	M+FA-H	C43H82NO8P	816.57	-0.88	HILIC_Neg
HMDB09296	PE(20:2(11Z,14Z)/20:1(11Z))	M+FA-H	C45H84NO8P	842.59	-0.47	HILIC_Neg
HMDB14732	Amiloride	M+Na	C6H8ClN7O	252.03	-1.56	HILIC_pos

Supplementary Figure S1

(A)

HILIC Positive

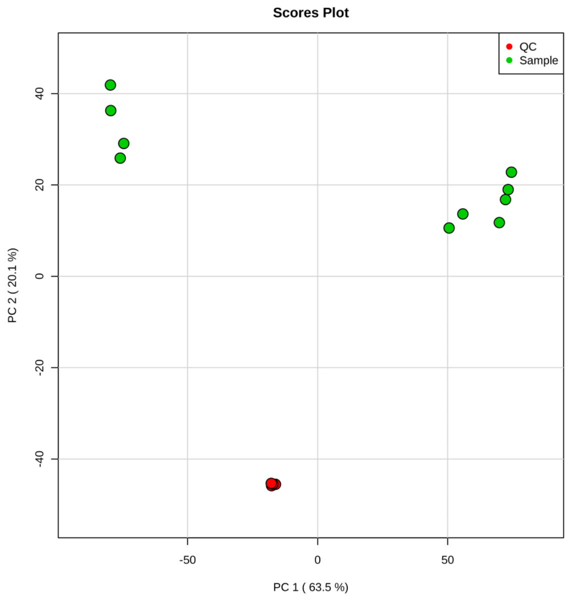

HILIC Negative

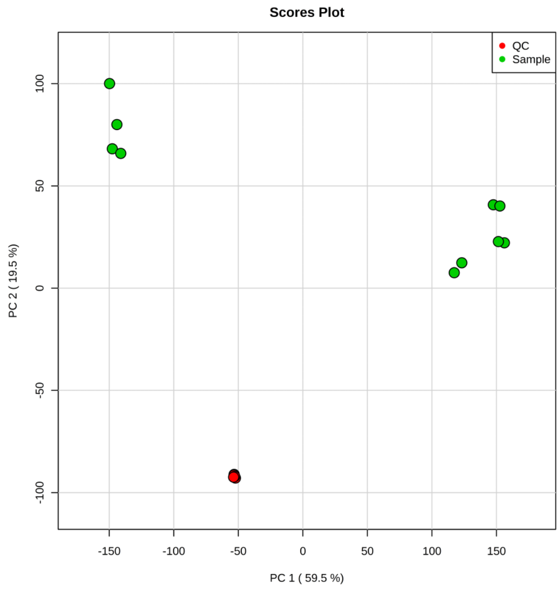

(B)

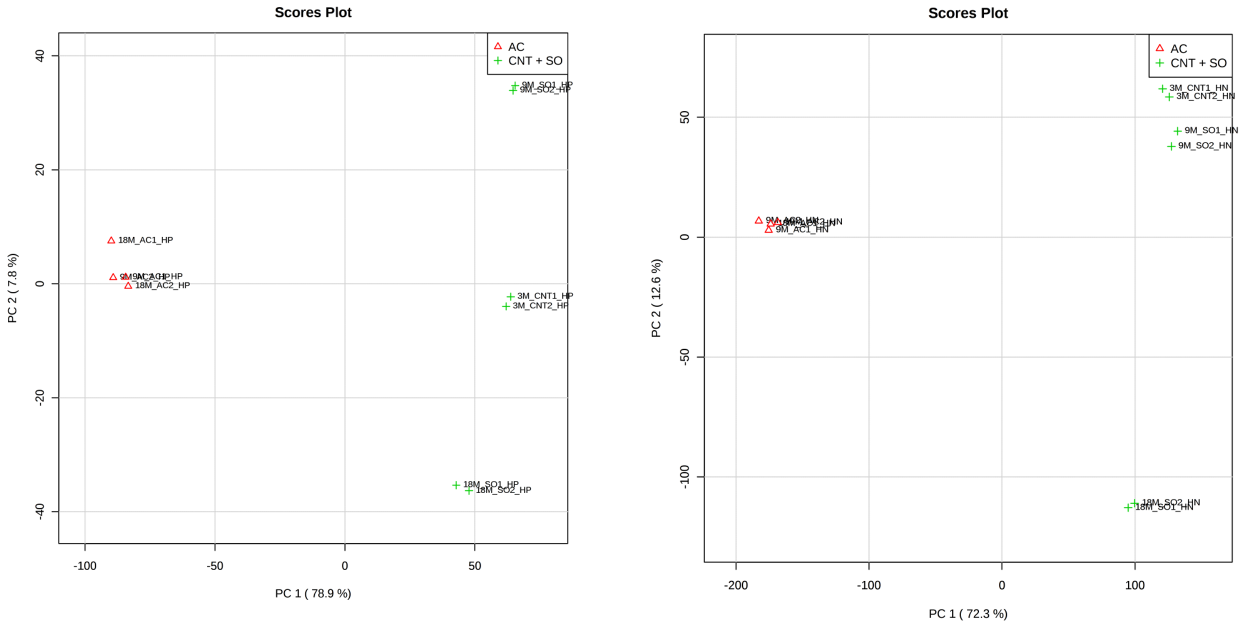

Supplementary Figure S1. Principle Component Analysis score plot obtained during metabolic profiling of heart tissues of 3 months old, sham operated and aorta constricted rats. (A) The principle component analysis (PCA) score plot of the quality control samples. Principal component analysis was done with Metaboanalyst 3.0. **(B)** Principal component analysis was done with Metaboanalyst 3.0. PCA analysis of control (CNT), Aorta constricted (AC), sham operated (SO) rats groups identified by LCMS in the positive and negative ion modes.

Supplementary Figure S2

A

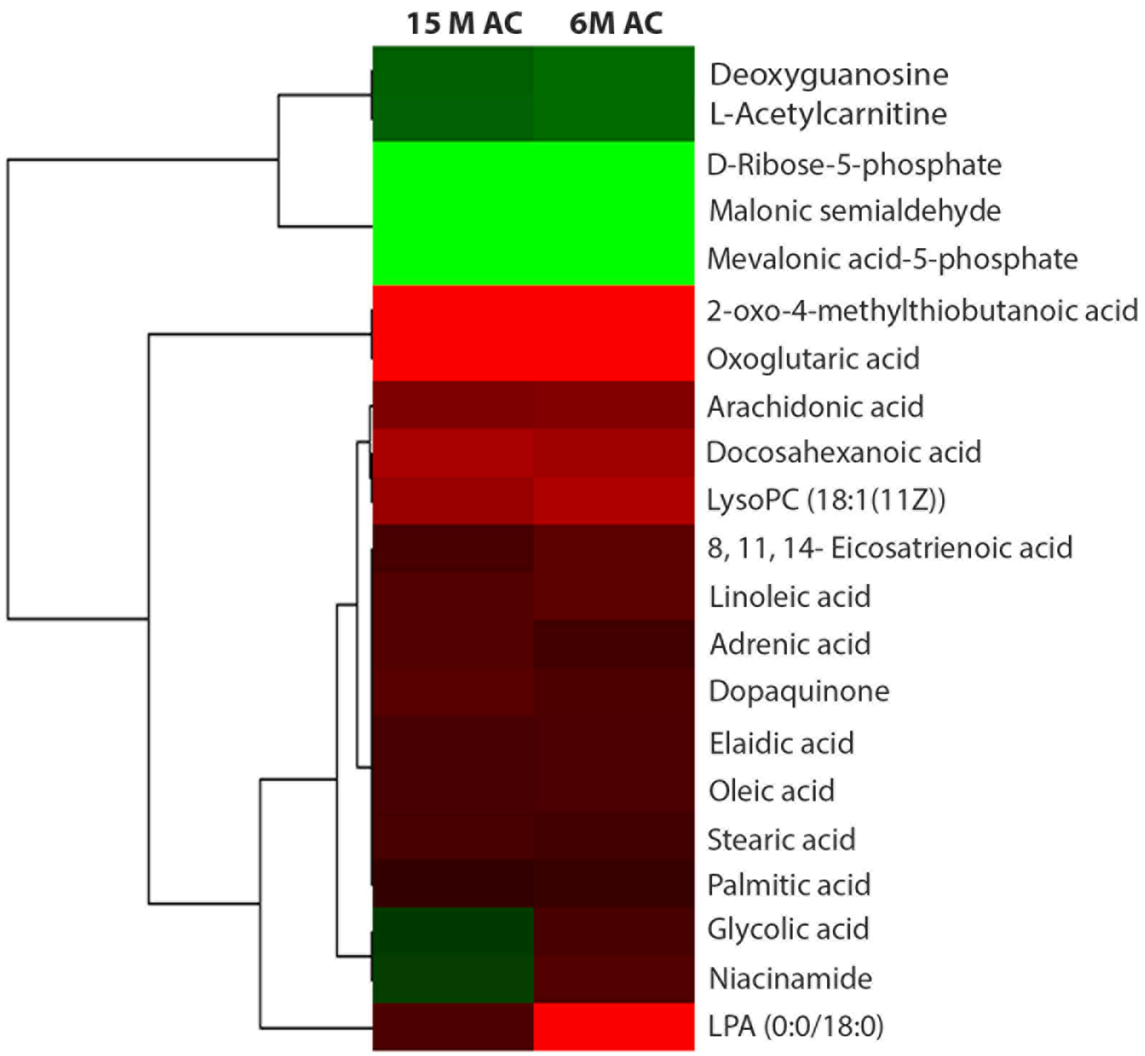
15 M AC
6M AC
Deoxyguanosine
L-Acetylcarnitine
D-Ribose-5-phosphate
Malonic semialdehyde
Mevalonic acid-5-phosphate
2-oxo-4-methylthiobutanoic acid
Oxoglutaric acid
Arachidonic acid
Docosahexanoic acid
LysoPC (18:1(11Z))
8, 11, 14- Eicosatrienoic acid
Linoleic acid
Adrenic acid
Dopaquinone
Elaidic acid
Oleic acid
Stearic acid
Palmitic acid
Glycolic acid
Niacinamide
LPA (0:0/18:0)

B

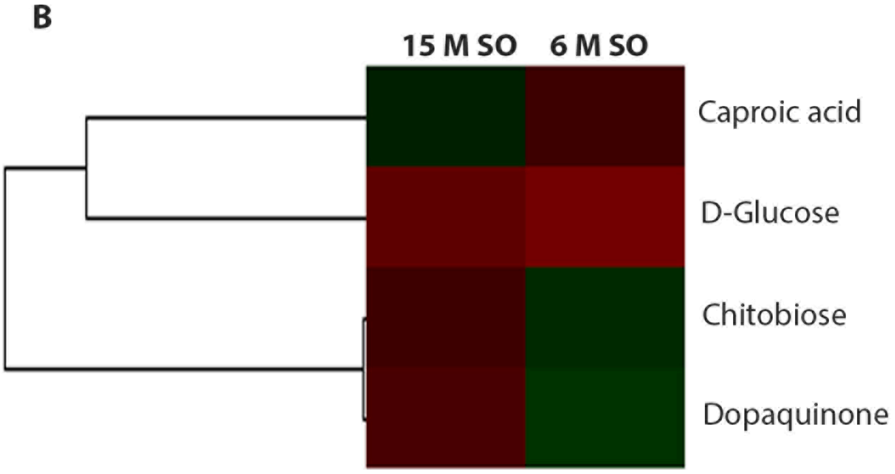
15 M SO
6 M SO
Caproic acid
D-Glucose
Chitobiose
Dopaquinone

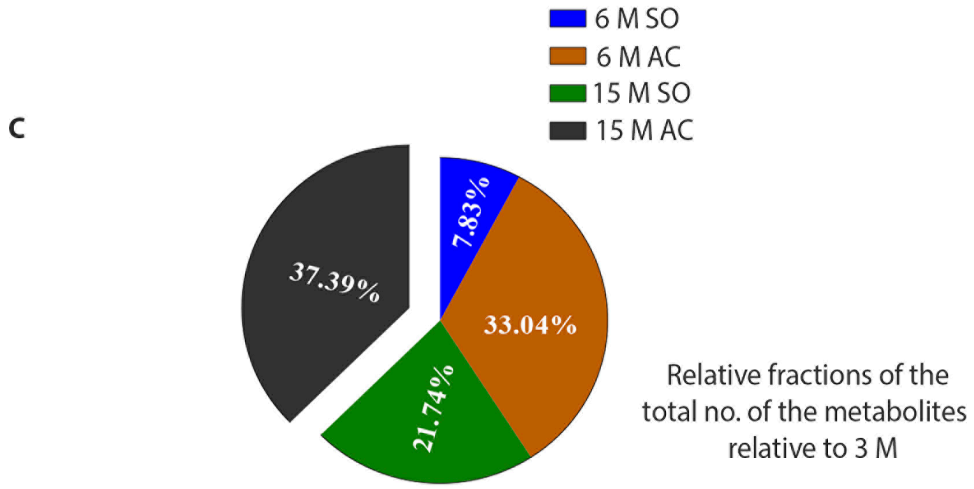

Supplementary Figure S2. **Heat map obtained from the metabolic data**. **(A)** Comparative presence of metabolites of left ventricular tissues from rats which had constriction of aorta compared to 3 months old rats. **(B)** Comparative presence of metabolites in left ventricle of heart tissue of rats which underwent sham operation compared to 3 months old rats. **(C)** Pie diagram represents the relative fraction of metabolites to the total number of metabolites (normalised to 3 M) present in heart tissue of 6 M SO/AC and 15 M SO/AC rats obtained after LC-MS analysis.

Supplementary Figure S3

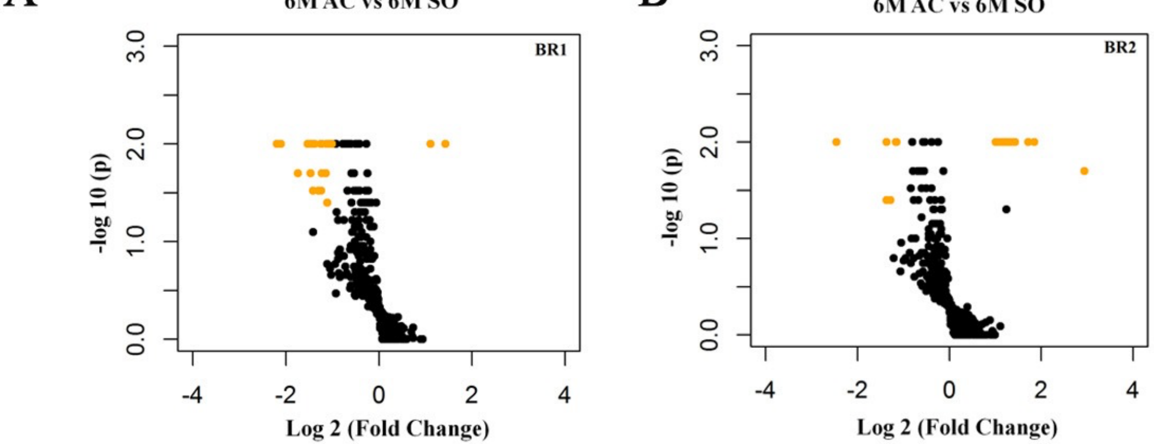

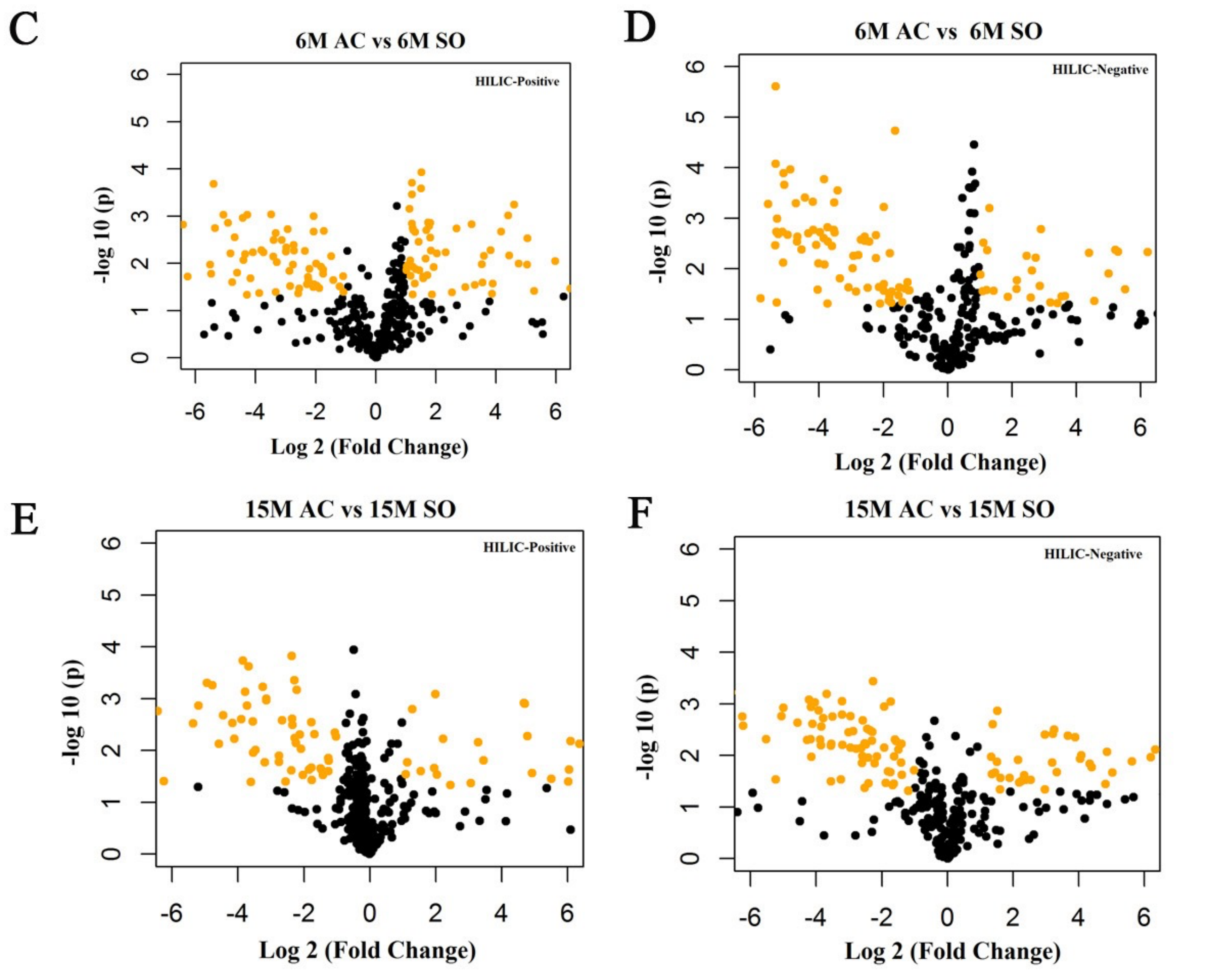

Supplementary Figure S3. Volcano plots represent the altered proteins and metabolites identified after LC-MS analysis. **(A, B)** Volcano plots represent the differential expressed proteins in heart tissues of rats from two different biological replicates, BR1 and BR2. **(C-F)** Volcano plots represents the metabolomics data acquired in HILIC positive ion mode and HILIC negative ion mode. The orange spots represent features that have a fold change > 2 and p-value < 0.05; Black spots represent the proteins or metabolites that have a fold change < 2 and p-value >0.05.

Supplementary Figure S4

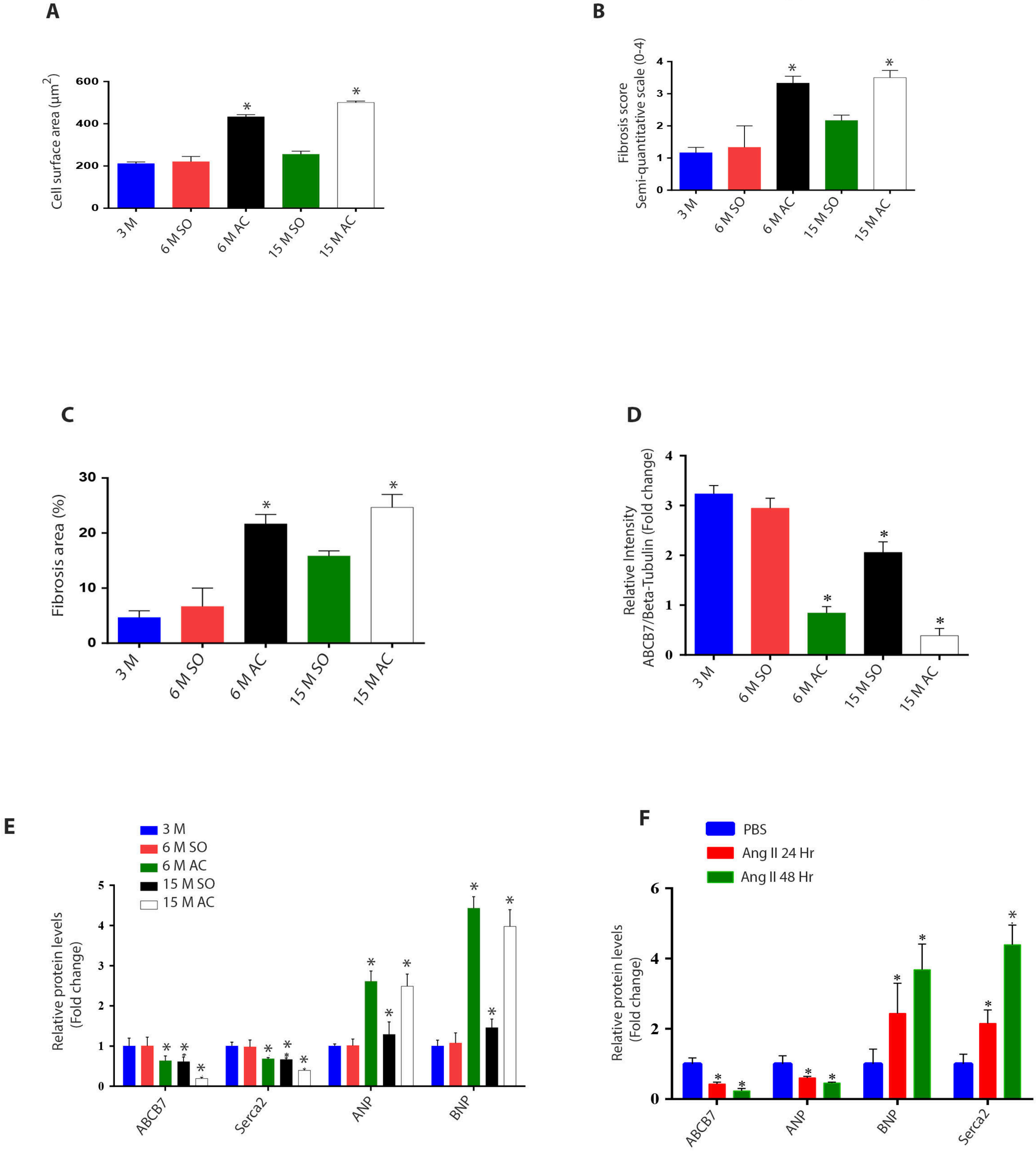

Supplementary Figure S4. (A) Quantification results of cross sectional area of WGA stained cardiomyocytes in heart tissues of rats which underwent either aorta constriction or sham operation or 3 M control rats as described in Fig. 1. **P*<0.05, AC vs 3 M/SO, n=8 rats in each group, ~100 cells per group. **(B, C)** Bar graphs for the quantitative results of fibrosis score and fibrosis area in cross sections of heart detected by trichrome staining as described in Fig. 1. **P*<0.05, AC vs 3 M/SO, n=8 rats in each group. **(D)** Bar graphs for the quantitative results of ABCB7 protein expression in heart tissues detected by immunohistochemistry as described in Fig. 3. **P*<0.05, AC vs 3 M/SO, n=8 rats in each group. **(E, F)** Bar graphs for the quantitative results of proteins ABCB7, ANP, BNP and Serca2 in heart tissue of rats (**P*<0.05, AC vs 3 M/SO, n=4 rats in each group) and H9C2 cells stimulated with Ang II (**P*<0.05, Ang II 24/48 hrs. vs PBS 24/48 hrs., n=3 independent experiments) as described in Fig. 3. ***P*<0.001, siABCB7 vs siRNA and Flag-ABCB7 vs Vector, n=3 independent experiments.

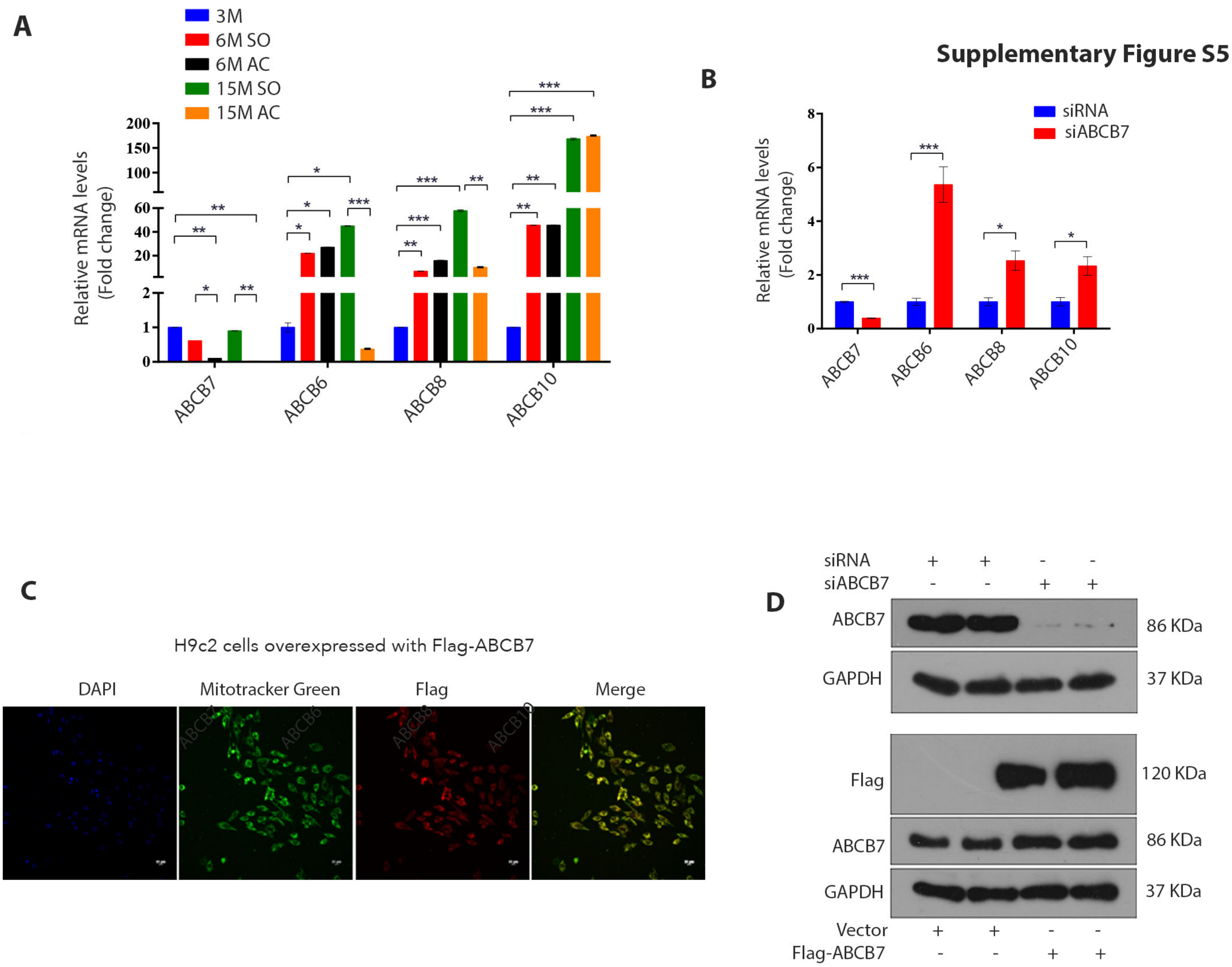

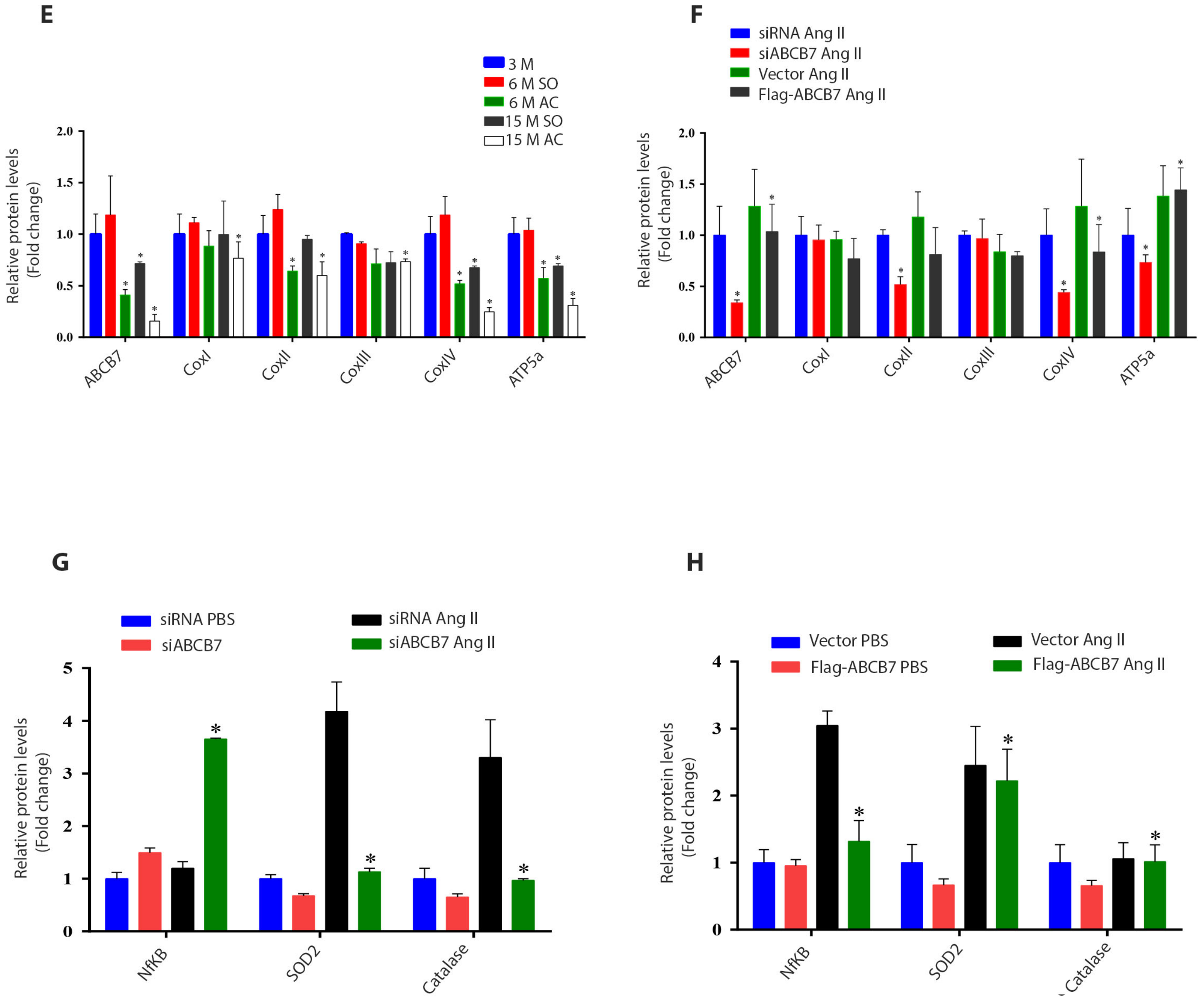

Supplementary Figure S5. **Expression of different ABCB transporters in heart tissues and H9c2 cells. (A, B)** mRNA expression analysis for ABCB6, ABCB7, ABCB8 and ABCB10 genes in the heart tissues of rats (**P*<0.05, AC vs 3 M and SO; n=4) and in ABCB7 silenced H9c2 cells respectively. **(C, D)** Expression of ABCB7 and flag in H9c2 cells transfected with Flag-ABCB7. **(E, F)** Bar graphs for the quantitative results of proteins CoxI, CoxII, CoxIII, CoxIV and ATP5a in heart tissue of rats (**P*<0.05, AC vs 3 M/SO, n=4 rats in each group) and H9C2 cells transfected with siABCB7 or Flag-ABCB7 under stimulation of And II. **(G, H)** Bar graphs for the quantitative results of NfkB, SOD2 and catalase proteins in H9C2 cells transfected with siABCB7 or Flag-ABCB7 under stimulation of And II. **P*<0.05, siABCB7 PBS/Ang II vs siRNA PBS/Ang II and Flag-ABCB7 PBS/Ang II vs Vector PBS/Ang II. n=3 independent experiments/group.

Supplementary Figure S6

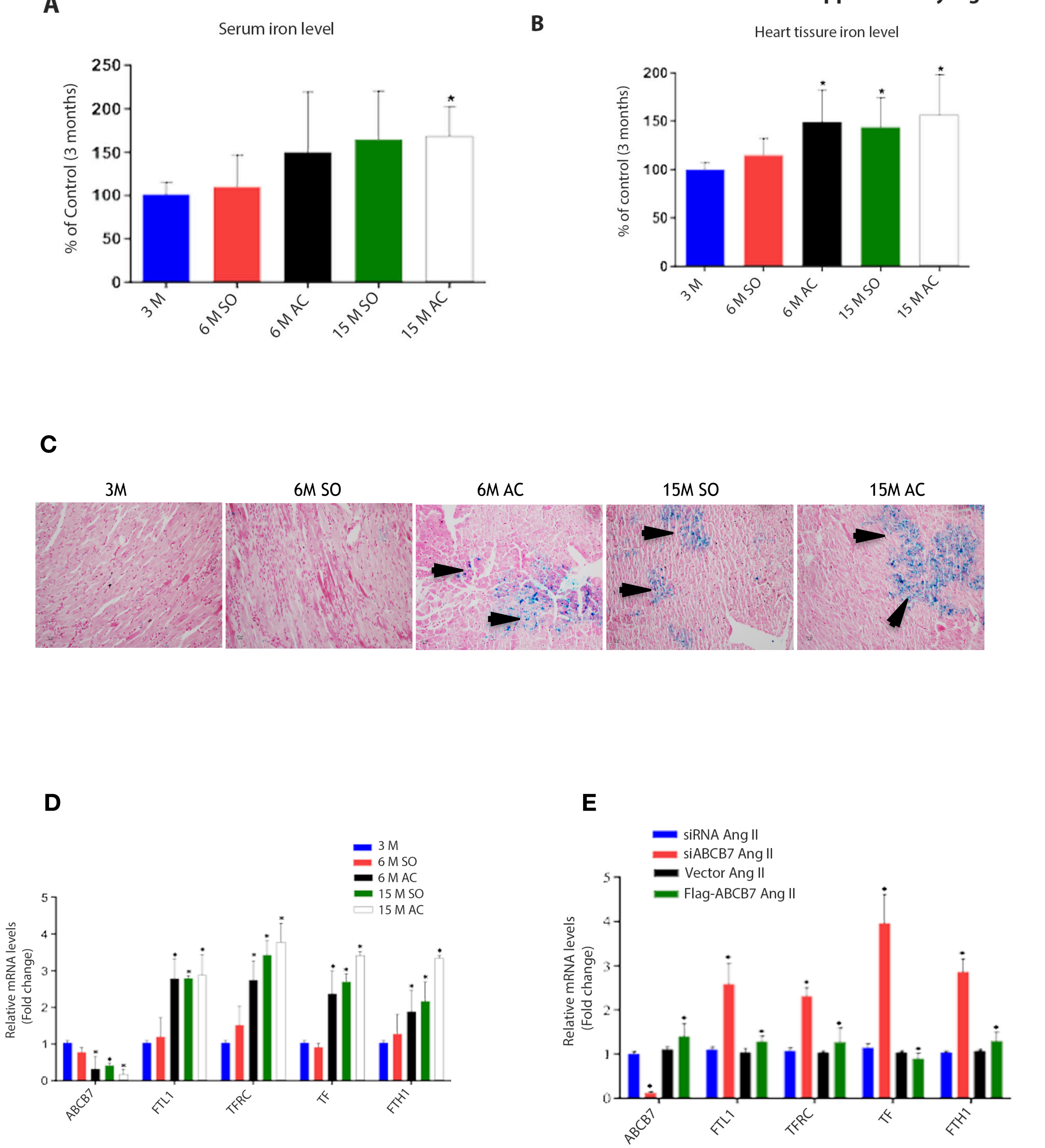

F

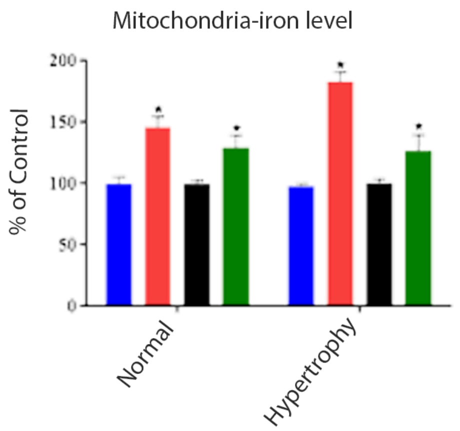

G

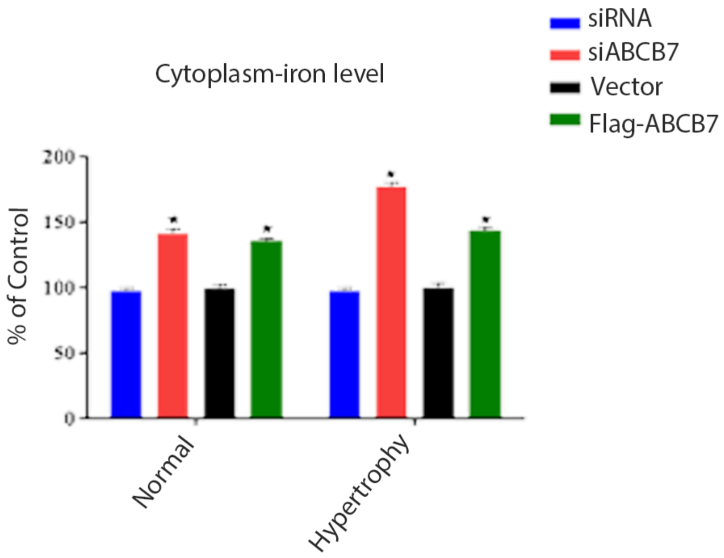

H

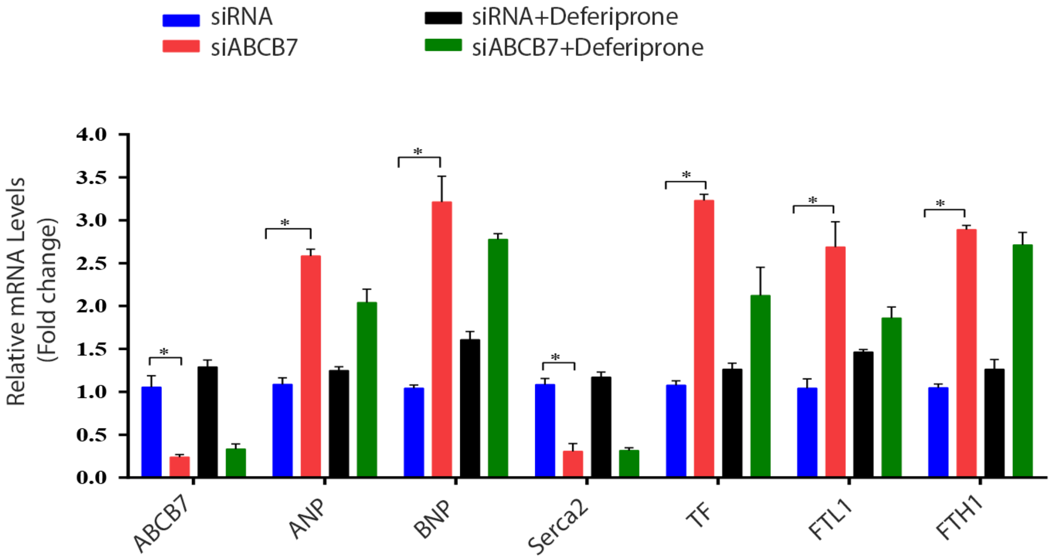

Supplementary Figure S6. **ABCB7 regulates cellular iron homeostasis in cardiomyocytes**. **(A, B)** Total iron content in heart tissue lysate and serum from 3 months old, sham operated and aorta constricted rats measured using iron assay kit (Abcam). *P<0.05, AC vs 3 M or SO; n=8. **(C)** Photomicrograph of Perl's Prussian blue stained heart tissues from 3 months old, sham operated and aorta constricted rats (20X). **(D, E)** Results of mRNA expression analysis for iron regulatory genes in heart tissues (*P<0.05, AC vs 3 M and SO; n=4) and H9C2 cells stimulated with angiotensin II after transfection with either siABCB7 or Flag-ABCB7 as determined using qRT-PCR. *P<0.05, siABCB7 vs control siRNA and vector vs flag-ABCB7; n=4. **(F, G)** Total iron content in mitochondrial and cytoplasmic fractions of H9C2 cells

stimulated with angiotensin II after transfection with either siABCB7 or Flag-ABCB7 measured using iron assay kit (Abcam). **(H)** mRNA levels of ABCB7, hypertrophy markers and iron regulatory genes in H9C2 cells treated with deferiprone (Fe^{3+} chelator) after transfection with either siABCB7 or Flag-ABCB7.**P*<0.05, siABCB7 vs control siRNA and vector vs flag-ABCB7; n=4. Data are expressed as mean±SEM. **P*<0.05, siABCB7 vs control siRNA and vector vs flag-ABCB7; n=4. Data are expressed as mean±SEM.

Supplementary Figure S7

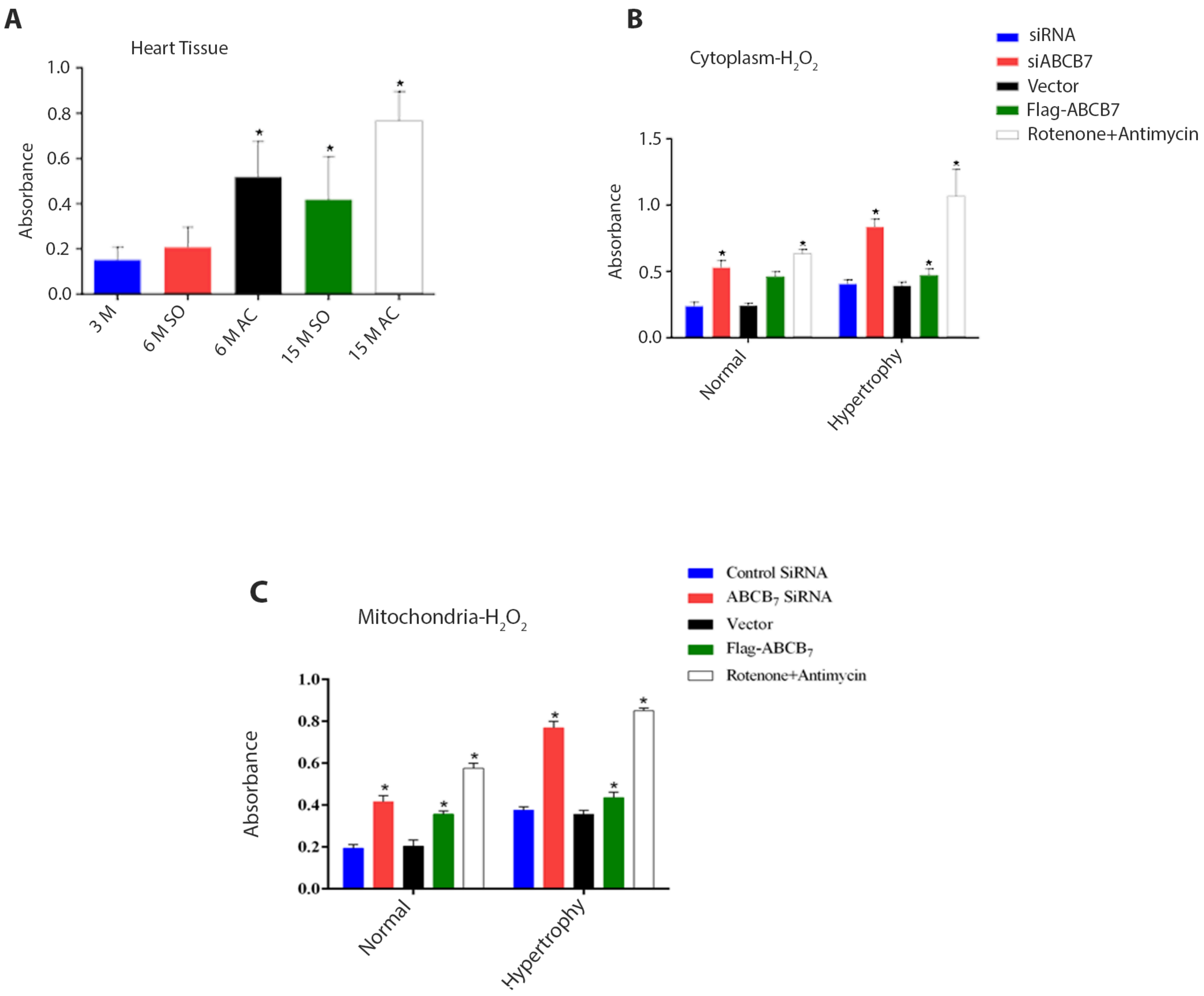

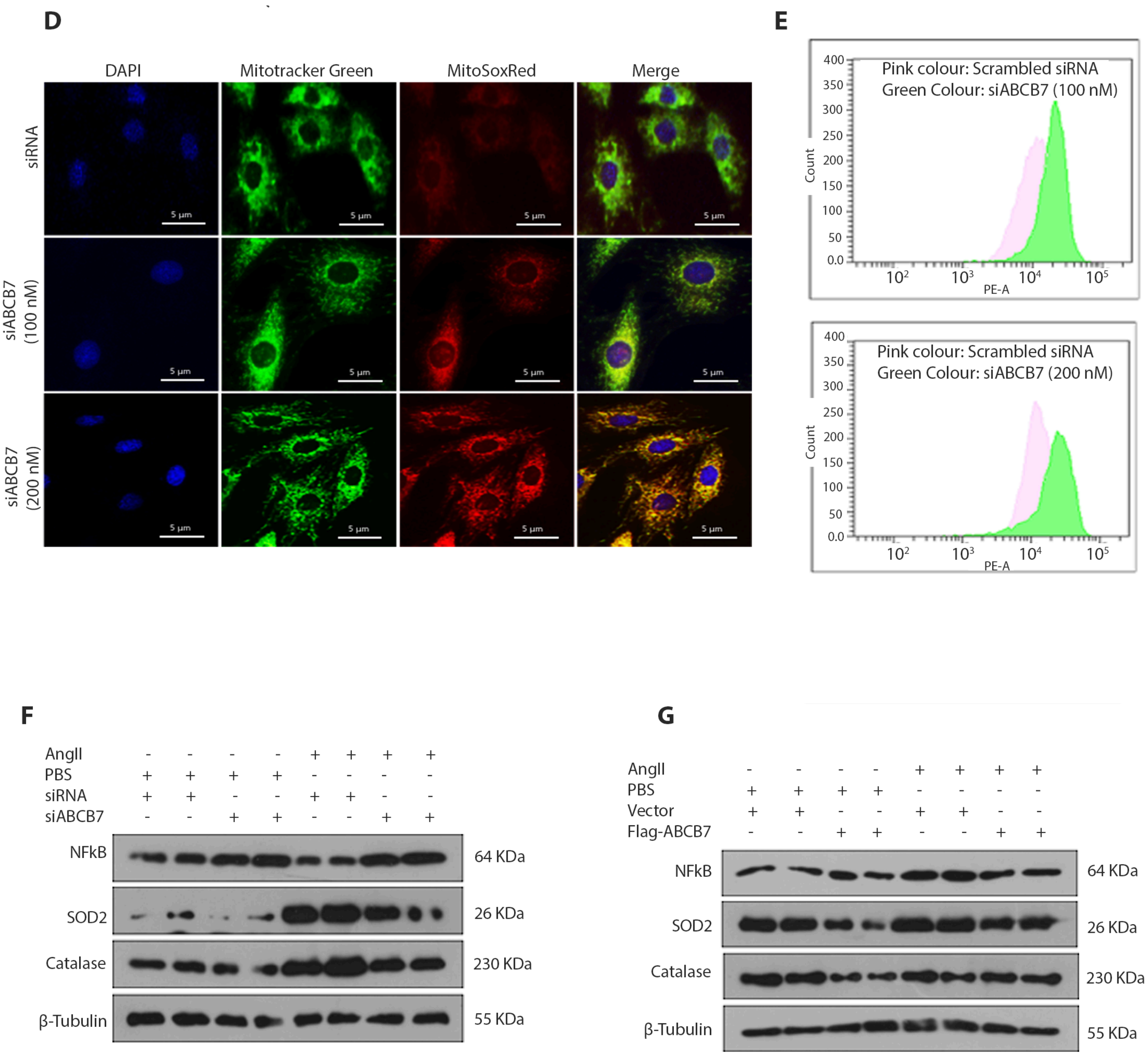

Supplementary Figure S7. ABCB7 deficiency increases ROS levels in cardiomyocytes. **(A)** Results of Amplex Red assay for H2O2 in freshly isolated mitochondria from heart tissue from 3 months old, sham operated and aorta constricted rats. **P*<0.05, AC vs 3 M and SO; n=8. **(B, C)** H2O2 levels from mitochondrial and cytoplasmic fractions from H9C2 cells stimulated with angiotensin II after transfection with either siABCB7 or flag-ABCB7. Rotenone and antimycin treated H9C2 cells were used as positive control. **P*<0.05, siABCB7 vs control siRNA and vector vs flag-ABCB7; n=4. **(D)** Immunofluorescent images of H9C2 cells transfected with different concentration of siABCB7 (100 nM and 200 nM) and stimulated with angiotensin II. An increase in MitoSOX Red uptake is seen in mitochondria of ABCB7 deficient cells indicating increase in mitochondrial ROS levels. **(E)** FACS analysis of ABCB7 deficient H9C2 cells revealing MitoSOX Red uptake in mitochondria of the cells. MitoSox Red positive cells are increased after knockdown of ABCB7 gene in H9C2 cells. **(F, G)** Representative immunoblots for NfkB, SOD2 and catalase expression in H9C2 cells transfected using either siABCB7 or flag-ABCB7 followed by stimulation with Angiotensin II or PBS for 24 hours. Data are expressed as mean±SEM.

Supplementary Figure S8

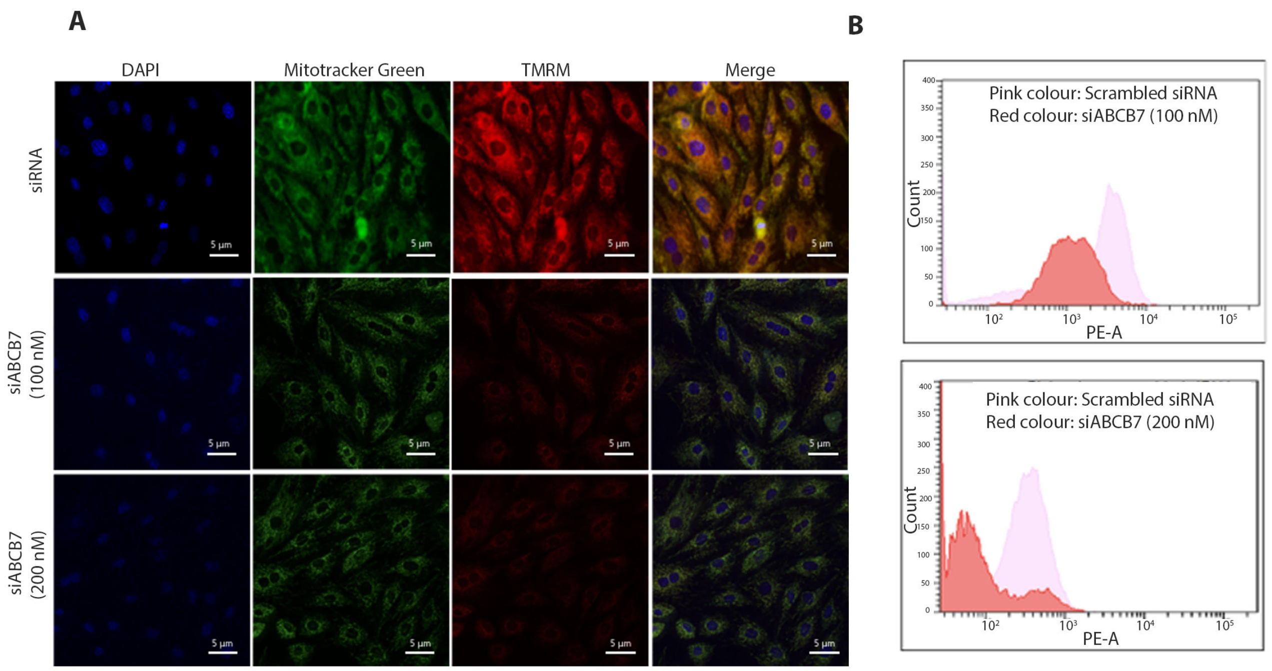

Supplementary Figure S8. ABCB7 deficiency impairs mitochondrial membrane integrity. Mitochondrial membrane potentials in H9C2 cells which were transfected with different concentrations of siABCB7 and measured using TMRM dye. **(A)** Confocal images reveal that intensity of red fluorescence directly relates to the uptake of TMRM dye by cells. **(B)** Quantification of TMRM positive cells by FACS analysis.

Supplementary Figure S9

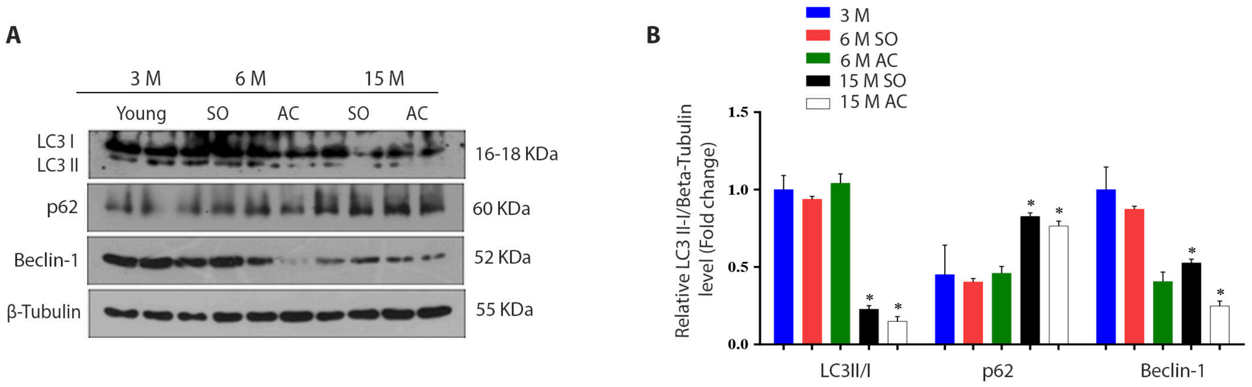

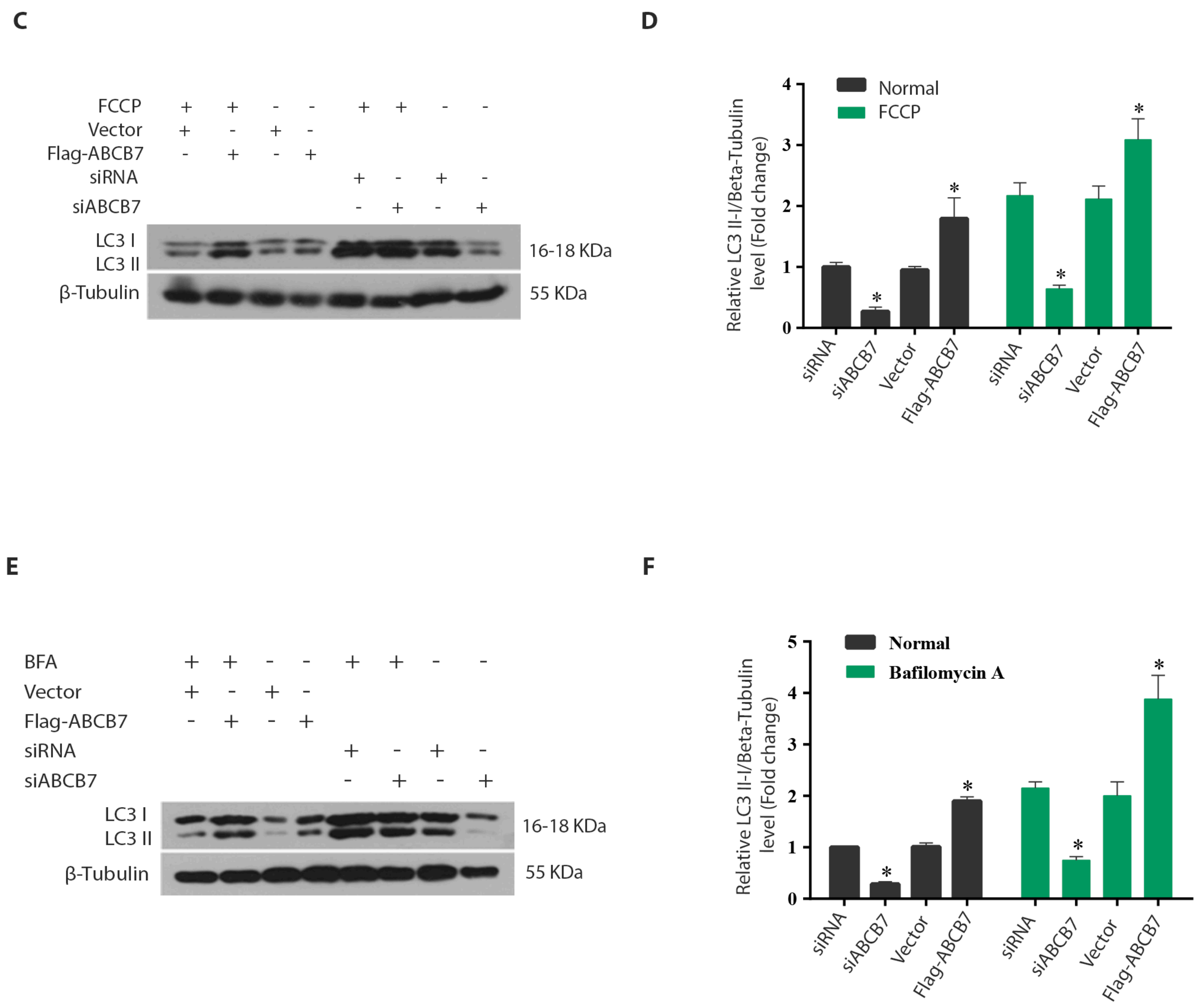

Supplementary Figure S9. ABCB7 deficiency contributes to impaired autophagy in hypertrophic cardiomyocytes. (A) Representative Western blots of lysates of heart tissues of rats with cardiac hypertrophy. Decreased LC3-I to LC3-II conversion in rats which had hypertrophy. **(C, E)** LC3I/II protein expression in H9C2 cells transfected using siABCB7 or control siRNA or with control vector or flag-ABCB7 followed by stimulation with FCCP or BFA or PBS for 24 hours. **(B, D, F)** Bar graphs for the quantitative results of LC3 II/I protein heart tissue of rats (**P*<0.05, AC vs 3 M/SO, n=4 rats in each group) and in H9C2 cells transfected with siABCB7 or Flag-ABCB7; treated with either FCCP or Bafilomycin A or PBS. **P*<0.05, siABCB7 PBS/FCCP/BFA vs siRNA PBS/FCCP/BFA and Flag-ABCB7 PBS/FCCP/BFA vs Vector PBS/FCCP/BFA. n=3 independent experiments/group.

Supplementary Figure S10

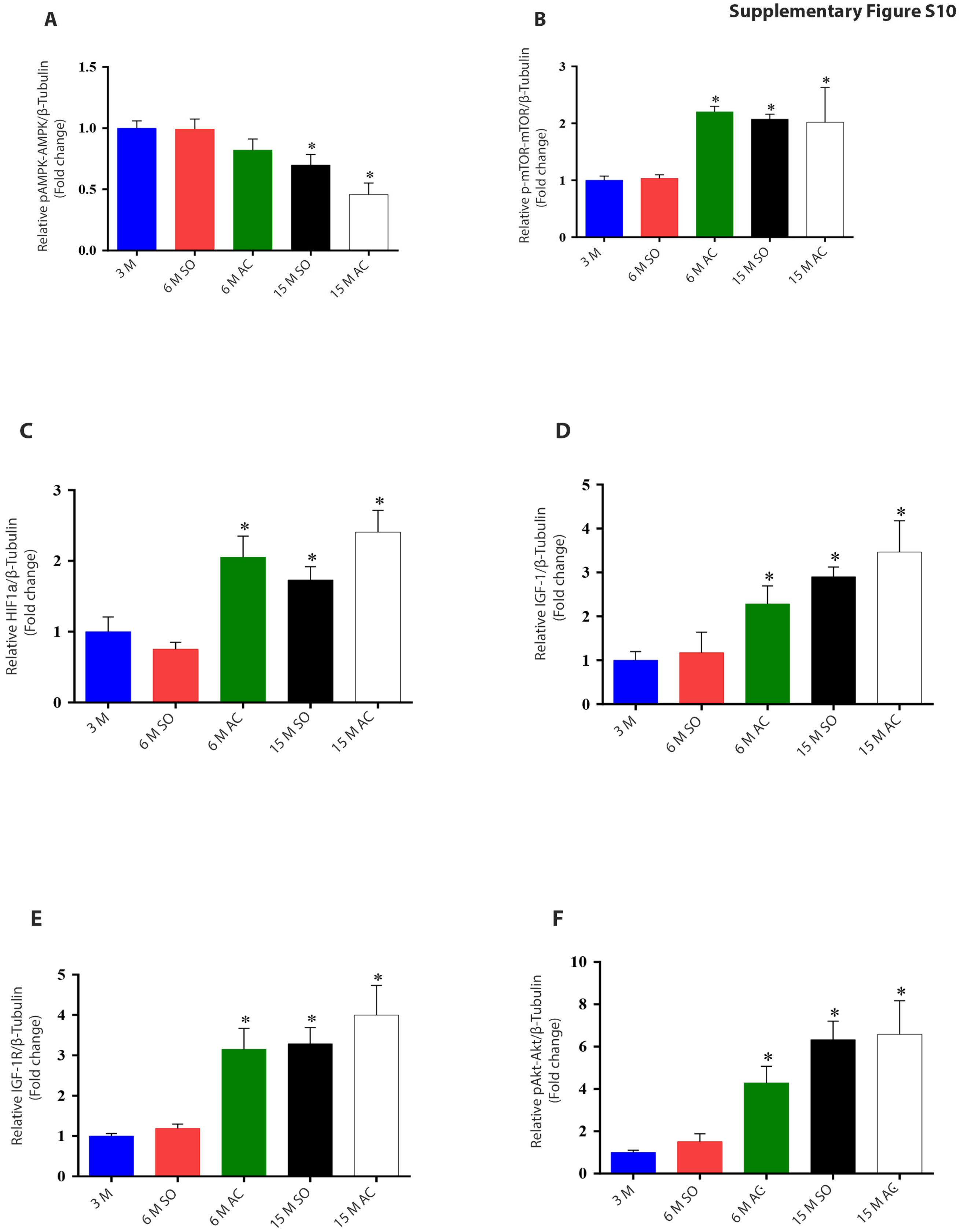

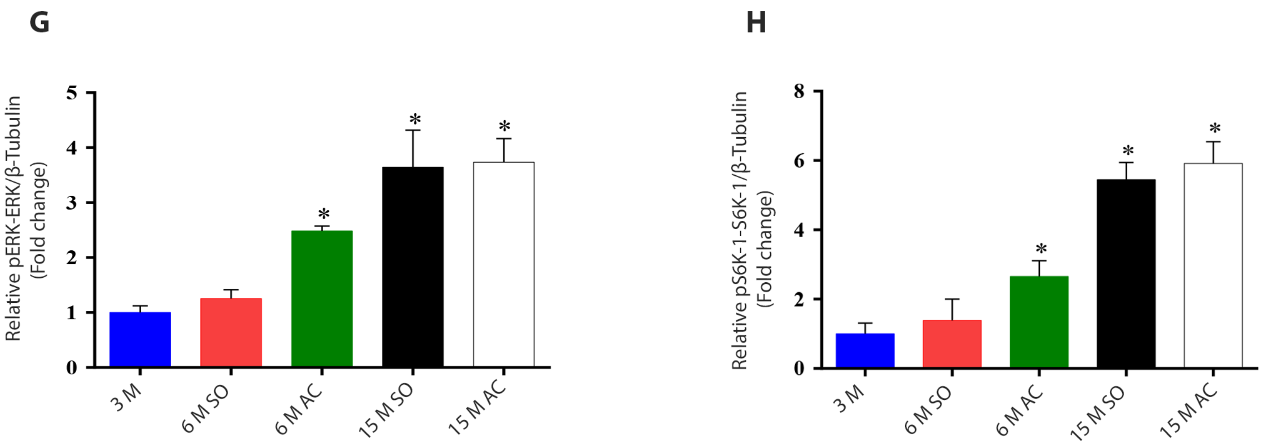

Supplementary Figure S10. Densitometric quantification results of protein bands detected by Immunoblotting as described in Fig. 9. (A-C) Bar graphs for the quantitative results of proteins pAMPK, p-mTOR and HIF1a in heart tissue of 3 M, 6 M and 15 M SO/AC rats. **P*<0.05, AC vs 3 M/SO, n=4 rats in each group. **(D-H)** Bar graphs for the quantitative results of proteins IGF-1, IGF1R, pAkt, pERK1/2 and p-S6K-1 in heart tissue of 3 M, 6 M and 15 M SO/AC rats. **P*<0.05, AC vs 3 M/SO, n=4 rats in each group.

Supplementary Figure S11

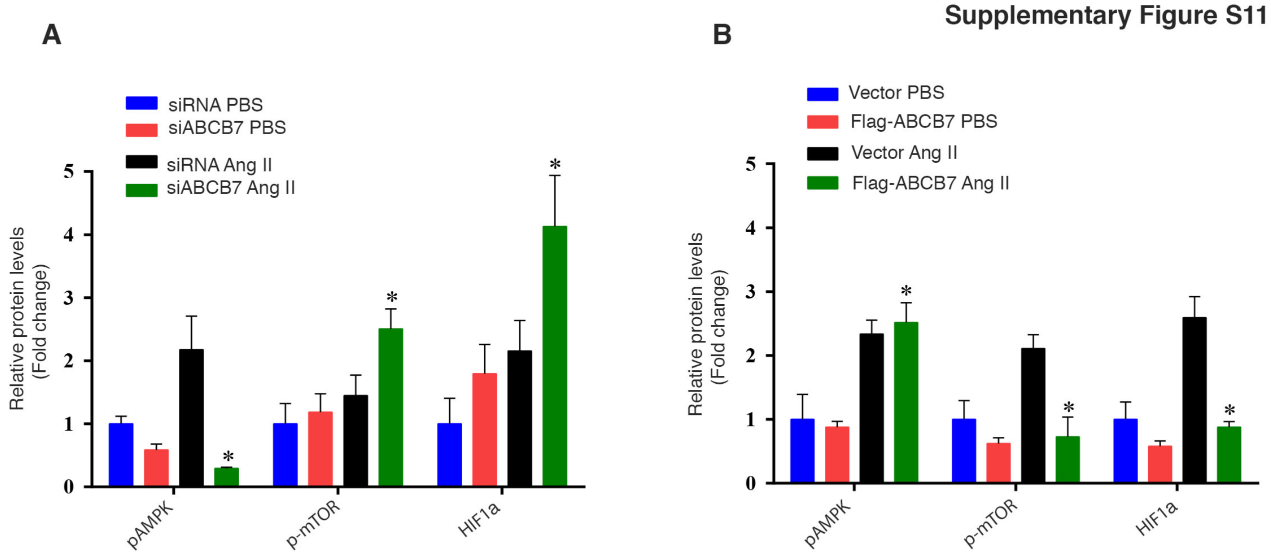

C

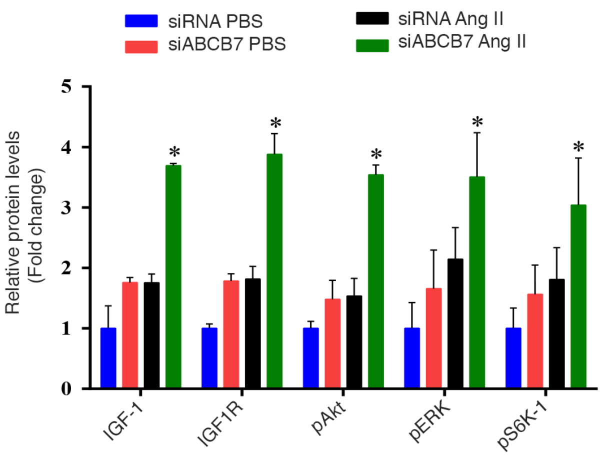
siRNA PBS
siABCB7 PBS
siRNA Ang II
siABCB7 Ang II
Relative protein levels
(Fold change)
0
1
2
3
4
5
IGF-1
IGF1R
pAkt
pERK
pS6K-1

D

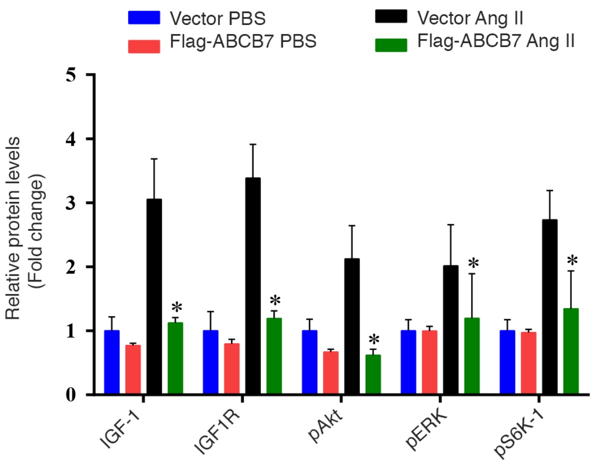
Vector PBS
Flag-ABCB7 PBS
Vector Ang II
Flag-ABCB7 Ang II
Relative protein levels
(Fold change)
0
1
2
3
4
5
IGF-1
IGF1R
pAkt
pERK
pS6K-1

E

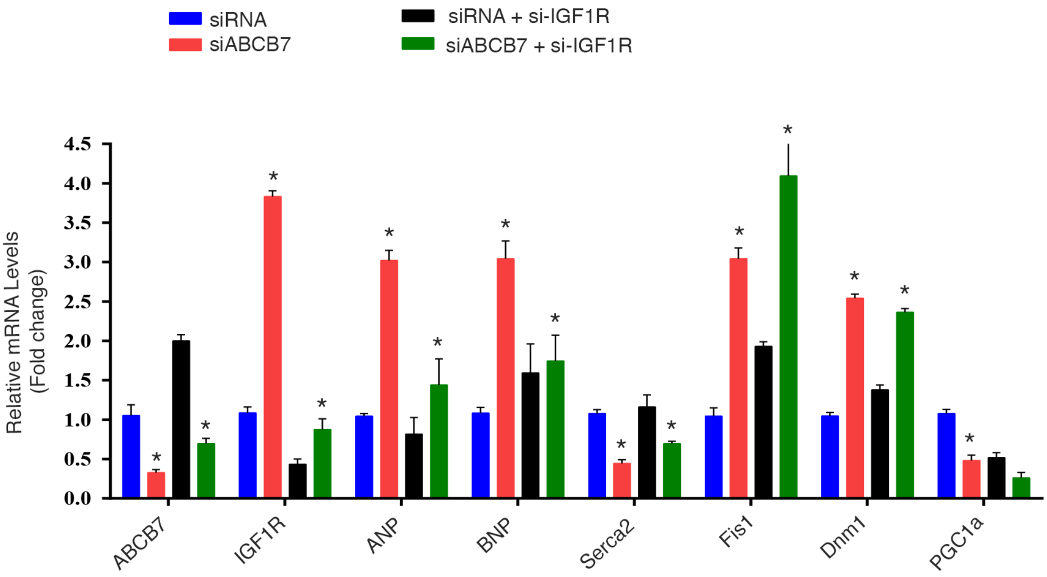
siRNA
siABCB7
siRNA + si-IGF1R
siABCB7 + si-IGF1R
Relative mRNA Levels
(Fold change)
0.0
0.5
1.0
1.5
2.0
2.5
3.0
3.5
4.0
4.5
ABCB7
IGF1R
ANP
BNP
Serca2
Fis1
Dnm1
PGC1a

F

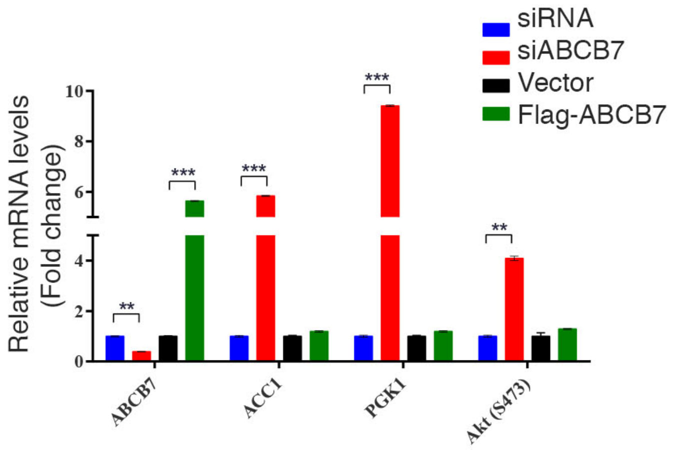

Supplementary Figure S11. Densitometric quantification results of protein bands detected by Immunoblotting as described in the manuscript. (A, B) Bar graphs for the quantitative results of proteins pAMPK, p-mTOR and HIF1a in H9C2 cells transfected with siABCB7 or Flag-ABCB7 under stimulation of And II. **P*<0.05, siABCB7 PBS/Ang II vs siRNA PBS/Ang II and Flag-ABCB7 PBS/Ang II vs Vector PBS/Ang II. n=3 independent experiments/group. **(C, D)** Bar graphs for the quantitative results of proteins IGF-1, IGF1R, pAkt, pERK1/2 and p-S6K-1 in H9C2 cells transfected with siABCB7 or Flag-ABCB7 under stimulation of And II. **(E)** mRNA levels of ABCB7, IGF1R, hypertrophy markers and mitochondrial dynamics genes in H9C2 cells silenced with si-IGF1R after transfection with either siABCB7 or Flag-ABCB7. **(F)** mRNA levels of ABCB7, ACC1, PGK1 and Akt (S473) genes in H9C2 cells transfected with either siABCB7 or Flag-ABCB7. **P*<0.05, siABCB7 PBS/Ang II vs siRNA PBS/Ang II and Flag-ABCB7 PBS/Ang II vs Vector PBS/Ang II. n=3 independent experiments/group.

Supplementary figure S12

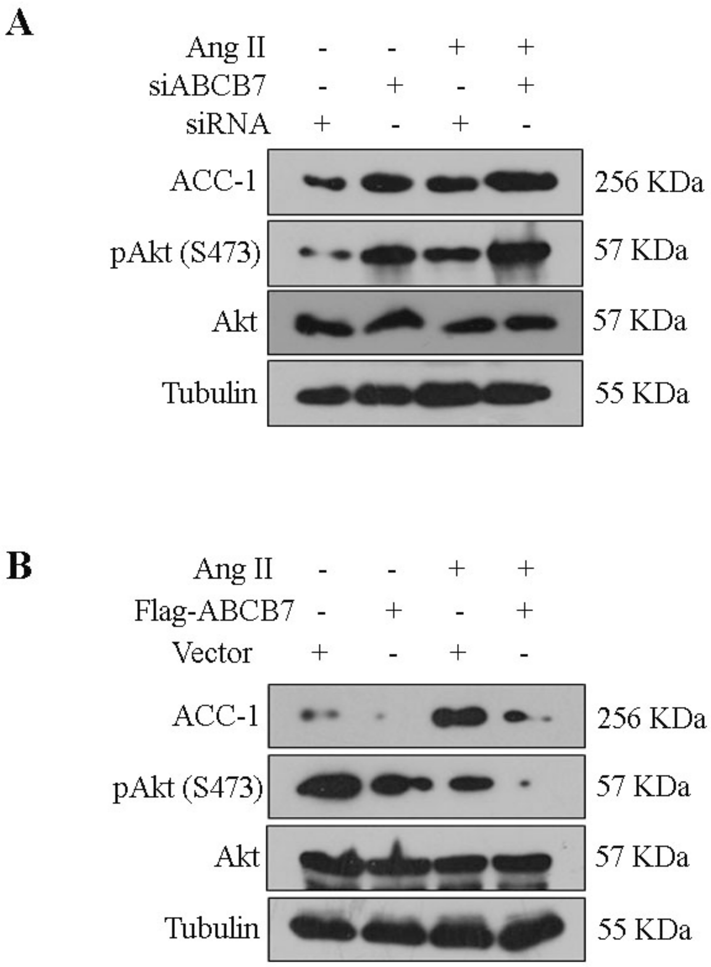

Supplementary Figure S12. ABCB7 deficiency is associated with enhanced expression of acetyl coA carboxylase (ACC1) and Akt (S473), the key AMPK and mTOR target proteins in hypertrophic H9c2 cells. (A, B) Western blot analysis of ACC1 and Akt (S473) proteins from hypertrophic H9C2 cells transfected with either siABCB7 or Flag-ABCB7.

Supplementary Table S7: List of antibodies used in the study.

Antibody	Company
ABCB7 (ATP binding cassette transporter B7)	Santacruz, Abcam
Wheat Germ Agglutinin (WGA)	Sigma-Aldrich
Atrial natriuretic peptide (ANP)	Santacruz
Brain natriuretic peptide (BNP)	Santacruz
Serca2	Cell signaling
Mitochondrial complex I (mt-CoxI)	Abcam
Mitochondrial complex II/SDHA (mt-CoxII)	Cell signaling, Abcam
Mitochondrial complex III (mt-CoxIII)	Cell signaling, Abcam
Mitochondrial complex IV (mt-CoxIV)	Cell signaling
Mitochondrial complex V (ATP5a)	Santacruz
LC3 I/II	Santacruz, Sigma-Aldrich
Superoxide dismutase (Sod2)	Cell signaling
Catalase	Abcam
NfKB	Santacruz
IGF-1R	Santacruz
IGF-1	Santacruz
ERK-1/pERK1	Cell signaling
Akt-1/pAkt-1	Santacruz, Cell signaling
pS6k-1	Cell signaling
AMPK/pAMPK	Cell signaling
p-mTOR	Cell signaling
HIF1a	Santacruz
β-Tubulin	Cell signaling
HSP60	Cell signaling
HSP70	Cell signaling
Flag	Abcam

Supplementary Table S8. List of primers used in the study.

Gene		Primer sequence
mt-Nd1	mt-Nd1 FP	5'-CCTCAACCTAGGCATACCATTT-3'
	mt-Nd1 RP	5'-AGGCTCATCCCGATCATAGA-3'
mt-Nd2	mt-Nd2 FP	5'-CCATTCTCGCAATTTCATCA-3'
	mt-Nd2 RP	5'-TTTCGTGTTTGGGTCTGGTT-3'
mt-Nd4	mt- Nd4 FP	5'-CATCAGTAAGCCATATAGCCCTAGTC-3'
	mt- Nd4 RP	5'-AAGCTTCATGGTGTCTGGATTAT-3'
mt-Co3	mt-Co3 FP	5'-TAAACCCAAGCCCATGACC-3'
	mt- Co3 RP	5'-AGCCGGATGTAAGTAGAAGAGC-3'
mt-Cytb	mt-Cytb FP	5'-CCCTAGTACTATTCTTCCCAGACCT-3'
	mt-Cytb RP	5'-AGGGGGTTAGCGGGTGTAT-3'
mt-Atp6	mt-Atp6 FP	5'-TAAGCATAGCCATCCCCCTA-3'
	mt-Atp6 RP	5'-TTAGTTTGTGTCGGAAGCCTAGA-3'
Cox5b	Cox5b FP	5'-GAATAGTGGGCTGCATCTGTG-3'
	Cox5b RP	5'-GGGCACCAACTTGTAATGTGT-3'
Opa1	Opa1 FP	5'-CTTTTGGCCAGCAAGGTTAG-3'
	Opa1 RP	5'-TCTTTCCATTCATCGAAGGTTT-3'
Drp1/Dnm1	Dnm1 FP	5'-TCCTGCAGCTGGTTAATTCT-3'
	Dnm1 RP	5'-CACTAGAGTCAGGTTGAGCACAT-3'
Fis1	Fis1 FP	5'-GAGCACGCAGTTTGAATACG-3'
	Fis1 RP	5'-ATTCCTTGAGCCGGTAGTTG-3'
FTH1	FTH1 FP	5'-CTGGAACTTCACAAACTGGCTA-3'
	FTH1 RP	5'-TCTTGCGTAAGTTGGTCACG-3'
FTL1	FTL1 FP	5'-ACACCTACCTCTCTCTGGGCTTC-3'
	FTL1 RP	5'-CGCGTTCGTTCTGCAACTTGA-3'
TFRC	TFRC FP	5'-AAGATGGATTTAGACCCAGCAG-3'
	TFRC RP	5'-ACCCAGGACGACTTTATCCA-3'
TF	TF FP	5'-CAGAAGGATTTATTTGGAAAGGTG-3'
	TF RP	5'-GTGGAAAGTGCAGGCTTCTAGGAG-3'
NRF1	NRF1 FP	5'-GTCCAAGATGCTAATGGCCCA-3'
	NRF1 RP	5'-TCCGTCTTCAACAACGTAAGCTCT-3'
TFAM	TFAM FP	5'-AAATTGCAGCCATGTGGAG-3'
	TFAM RP	5'-TGACTTGGAGTTAGCTGCTCTTT-3'
ABCB7	ABCB7 FP	5'-TATGTGTGGCCCAAAGACAG-3'

	ABCB7 RP	5’-TTGAGGCTGTCTACAGCATATTT-3’
ABCB7 (primer for cloning)	ABCB7c FP	5’-AAAAAGCTTCCCTCGCTCAAGATG-3’
	ABCB7c RP	5’-AAAGAATTCAACCAAACAAGACAACAA-3’
Slc25a37	Slc25a37 FP	5’-TATGAGAACCTACCGACCAGTG-3’
	Slc25a37 RP	5’-CTTTGGGATCTGGGTTCAAA-3’
Slc25a28	Slc25a28 FP	5’-TTGCCTGCTACGAAAAGTTAAA-3’
	Slc25a28 RP	5’-AAGTAACGTTGCCACACATCC-3’
PGC1α	PparGc1a FP	5’-ACATCGCAATTCTCCCTTGT-3’
	PparGc1a RP	5’-TTGAGCCTTTCGTGCTCAT-3’
Mfn1	Mfn1 FP	5’-AGATAATGCAGCCCAGGAAG-3’
	Mfn1 RP	5’-ATGAGTTTCCAGCCCACTGT-3’
Mfn2	Mfn2 FP	5’-ATGATCAGGTTCAGCGTCCT-3’
	Mfn2 RP	5’-ACGAGAATGCCCATAGAGGT-3’
ANP	ANP FP	5’-GTATACAGTGCGGTGTCCAA-3’
	ANP RP	5’-ATCTCTCTGAGACGGGTTGACT-3’
BNP	BNP FP	5’-AACAATCCACGATGCAGAAG-3’
	BNP RP	5’-GTGCCATCTTGGAATTTCG-3’
SERCA2	SERCA2 FP	5’-TTCGAAGTCTGCCTTCTGTG-3’
	SERCA2 RP	5’-CTCCAATGGGTGCATAGGTT-3’
ABCB6	ABCB6 FP	5’-CCTGACATCATTCTGCTGGA-3’
	ABCB6 RP	5’-TCTCTATGATGCAGCCATCC-3’
ABCB8	ABCB8 FP	5’-CCGATTTGGGAAGCTAGATG-3’
	ABCB8 RP	5’-ATCAACACTGTGGGCCTCTT-3’
ABCB10	ABCB10 FP	5’-AGAGGATCGCAATAGCCAGA-3’
	ABCB10 RP	5’-GTTAAGGACGGCAACGAAAT-3’
ACC1	ACC1 FP	5’-ACATCCCGCACCTTCTTCTA-3’
	ACC1 RP	5’-GCCATTCCACCAGATCCTTA-3’
PGK1	PGK1 FP	5’-ATTGTTTGGAACGGTCCTGT-3’
	PGK RP	5’-GTGCTCACATGGCTGACTTT-3’
